# A Multimodal Multi‐Shank Fluorescence Neural Probe for Cell‐Type‐Specific Electrophysiology in Multiple Regions across a Neural Circuit

**DOI:** 10.1002/advs.202103564

**Published:** 2021-11-19

**Authors:** Namsun Chou, Hyogeun Shin, Kanghwan Kim, Uikyu Chae, Minsu Jang, Ui‐Jin Jeong, Kyeong‐Seob Hwang, Bumjun Yi, Seung Eun Lee, Jiwan Woo, Yakdol Cho, Changhyuk Lee, Bradley J. Baker, Soo‐Jin Oh, Min‐Ho Nam, Nakwon Choi, Il‐Joo Cho

**Affiliations:** ^1^ Center for BioMicrosystems, Brain Science Institute Korea Institute of Science and Technology 5, Hwarang‐ro 14‐gil, Seongbuk‐gu Seoul 02792 Republic of Korea; ^2^ School of Electrical Engineering Korea University 145 Anam‐ro, Seongbuk‐gu Seoul 02841 Republic of Korea; ^3^ School of Mechanical Engineering Yonsei University 50 Yonsei‐ro, Seodaemun‐gu Seoul 03722 Republic of Korea; ^4^ Center for Functional Connectomics Brain Science Institute Korea Institute of Science and Technology 5, Hwarang‐ro 14‐gil, Seongbuk‐gu Seoul 02792 Republic of Korea; ^5^ Virus Facility, Research Animal Resource Center Brain Science Institute Korea Institute of Science and Technology 5, Hwarang‐ro 14‐gil, Seongbuk‐gu Seoul 02792 Republic of Korea; ^6^ Center for Neuroscience, Brain Science Institute Korea Institute of Science and Technology 5, Hwarang‐ro 14‐gil, Seongbuk‐gu Seoul 02792 Republic of Korea; ^7^ KU‐KIST Graduate School of Converging Science and Technology Korea University 145 Anam‐ro, Seongbuk‐gu Seoul 02841 Republic of Korea; ^8^ Division of Bio‐Medical Science and Technology, KIST School Korea University of Science and Technology 5, Hwarang‐ro 14‐gil, Seongbuk‐gu Seoul 02792 Republic of Korea; ^9^ School of Electrical and Electronics Engineering Yonsei University 50 Yonsei‐ro, Seodaemun‐gu Seoul 03722 Republic of Korea; ^10^ Yonsei‐KIST Convergence Research Institute Yonsei University 50 Yonsei‐ro, Seodaemun‐gu Seoul 03722 Republic of Korea

**Keywords:** cell‐type identification, electrophysiology, fluorescence signal, genetically encoded indicator, neural circuit, neural probe

## Abstract

Cell‐type‐specific, activity‐dependent electrophysiology can allow in‐depth analysis of functional connectivity inside complex neural circuits composed of various cell types. To date, optics‐based fluorescence recording devices enable monitoring cell‐type‐specific activities. However, the monitoring is typically limited to a single brain region, and the temporal resolution is significantly low. Herein, a multimodal multi‐shank fluorescence neural probe that allows cell‐type‐specific electrophysiology from multiple deep‐brain regions at a high spatiotemporal resolution is presented. A photodiode and an electrode‐array pair are monolithically integrated on each tip of a minimal‐form‐factor silicon device. Both fluorescence and electrical signals are successfully measured simultaneously in GCaMP6f expressing mice, and the cell type from sorted neural spikes is identified. The probe's capability of combined electro‐optical recordings for cell‐type‐specific electrophysiology at multiple brain regions within a neural circuit is demonstrated. The new experimental paradigm to enable the precise investigation of functional connectivity inside and across complex neural circuits composed of various cell types is expected.

## Graphical abstract

A multimodal multi‐shank fluorescence neural probe allows cell‐type‐specific electrophysiology from multiple deep‐brain regions at a high spatiotemporal resolution. The tip of probe monolithically integrates photodiode and an electrode‐array pair to measure both fluorescence and electrical signals simultaneously. The probe enables combined electro‐optical recordings for cell‐type‐specific electrophysiology at multiple brain regions within a neural circuit.

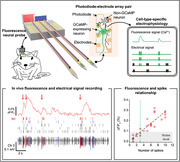

## 1 Introduction

Understanding the functions of particular regions of the brain and determining the causes of brain diseases require an intensive study of the target neural circuits. For the investigation of neural circuits, neuroscientists traditionally have used electrodes to measure electrical signals that represent neuronal activities from different areas of the brain, including local field potential and multi‐unit or single‐unit neural activity.^[^ ^1^, ^2^, ^3^, ^4^, ^5^ ^]^ Recent technological advances in neural electrode technology, including the development of multi‐shank neural probes and/or large‐channel‐count neural probes, have enabled the simultaneous, large‐scale measurement of single‐unit activities in different parts of a neural circuit.^[^ ^6^, ^7^, ^8^, ^9^ ^]^ Furthermore, analysis of the timing differences between single‐unit activities enables the identification of the connections between the cells.^[^ ^10^, ^11^ ^]^ However, a complete understanding of the advanced functions of the neural circuits requires the additional identification of the types of cells (e.g., activity: excitatory and inhibitory; neurotransmitter: dopaminergic,^[^ ^12^, ^13^ ^]^ GABAergic,^[^ ^14^, ^15^, ^16^ ^]^ glutamatergic,^[^ ^15^, ^16^, ^17^ ^]^ and others) in specific signal propagation pathways. Therefore, the study of neural circuits using the monitoring of cell‐type‐specific activity has contributed significantly to revealing the functional connections that exist between the regions of the brain.^[^ ^12^, ^13^, ^14^, ^15^, ^16^, ^17^ ^]^ Through this approach, recent studies have identified the mechanism of neurological diseases^[^ ^15^, ^16^, ^18^ ^]^ and developed treatment methods.^[^ ^12^, ^13^, ^17^ ^]^

The utilization of genetically encoded fluorescent indicators (GEFIs) has been dominant^[^ ^19^, ^20^, ^21^, ^22^ ^]^ for recording cell‐type‐specific neuronal activities because optical monitoring allows, in principle, spatiotemporal mapping. Accordingly, a variety of optical tools using microscopes,^[^ ^22^, ^23^, ^24^ ^]^ GRIN lenses,^[^ ^22^, ^25^, ^26^ ^]^ fibers,^[^ ^22^, ^27^, ^28^, ^29^ ^]^ and photometers^[^ ^30^, ^31^ ^]^ have been developed to record fluorescence signals that change in response to a variety of indicators of cellular activity (e.g., intracellular voltage,^[^ ^19^, ^20^ ^]^ calcium level,^[^ ^19^, ^20^ ^]^ and neurotransmitter concentration^[^ ^20^, ^21^ ^]^). The optical techniques are excellent candidates, complementary to electrophysiology to enable the identification of the activity of specific types of cells. While the optical methods enabled the monitoring of cell‐type‐specific activities, the temporal resolution is still significantly low, making it challenging to investigate neural circuits at the cellular level. Thus, simultaneous monitoring of electrical and fluorescent neural signals has been almost inevitable. However, most of these techniques are either inherently impossible or non‐trivial to integrate due to bulkier size limits toward multi‐channel extracellular electrophysiology supporting the simultaneous recording of multimodal activity.

Recently, a few optical fiber‐like devices assembled with electrodes have been developed^[^ ^32^, ^33^ ^]^ so that electrophysiology can be conducted in parallel with fiber photometry. However, these systems could record electrical signals from only a few neurons near the tip of the fiber using a single electrode. In addition, the complicated assembly process and bulky packaging make it difficult to implement an array structure which is essential for monitoring neural circuits at multiple brain regions. An ideal device for cell‐type‐specific analysis of neural circuits at multiple brain regions with high‐spatiotemporal‐resolution ought to meet the following requirements:

(1) The device must include fluorescence sensors for the detection of activity by specific type of cells.
(2) The device must have high‐density electrode arrays for high‐temporal‐resolution detection of electrical activity.
(3) The fluorescence sensors and the electrode arrays must exist in tightly integrated pairs for the co‐localized recording of the activities.
(4) Multiple pairs of these devices must be integrated as an array structure into a single platform at pre‐determined locations to support precise targeting of the multiple regions within a neural circuit.

Herein, we present a multimodal multi‐shank neural probe that is monolithically integrated with 1) photodiode for the measurement of cell‐type‐specific fluorescent signal, 2) electrode arrays for recording neural signal, and 3) optical fibers and LEDs for the delivery of excitation light. The tightly packed, photodiode‐electrode‐array pairs on each silicon shank are crucial because this configuration allows the measurement of multimodal signals from individually identical neurons located near the photodiode. This simultaneous recording of concurrent fluorescence and electrical signals enables the cell‐type‐specific electrophysiology, thereby allowing the study of the role of specific cell types in neural circuits. Also, the multi‐shank structure enables the recording of multimodal neural signals from different regions of the brain, which is essential for the study of neural circuits. To demonstrate the functionality of the probe, we measured bimodal signals in the hippocampal CA1 region of Syn‐GCaMP6f expressed mice and successfully matched the fluorescence signals with neural spikes. We then demonstrated the probe's capability of the multi‐regional cell‐type‐specific electrophysiology through recordings of multimodal signals from multiple locations inside a brain circuit. With multi‐shank probes implanted into the brain of mice expressing CaMKII*α*‐GCaMP6f in the hippocampus, we measured electrical signals of neurons in multiple locations inside the CA1 and successfully identified the excitatory neurons with the fluorescence signal. An additional Syn‐ChrimsonR expression in the CA3 region and the optical stimulation provided to the region through an additional shank of the probe assisted the study with increased activities in the CA1 region. The results presented in this work show that the fluorescence neural probe can be used 1) to identify the roles of a specific type of cells through cell‐type‐specific electrophysiology, 2) to study the functional connectivity of specific types of cells, and 3) to study a wide range of neural circuits composed of a specific type of cells.

## 2 Results

### 2.1 Design, Fabrication, and Packaging of the Multimodal Fluorescence Neural Probe

**Figure 1:**
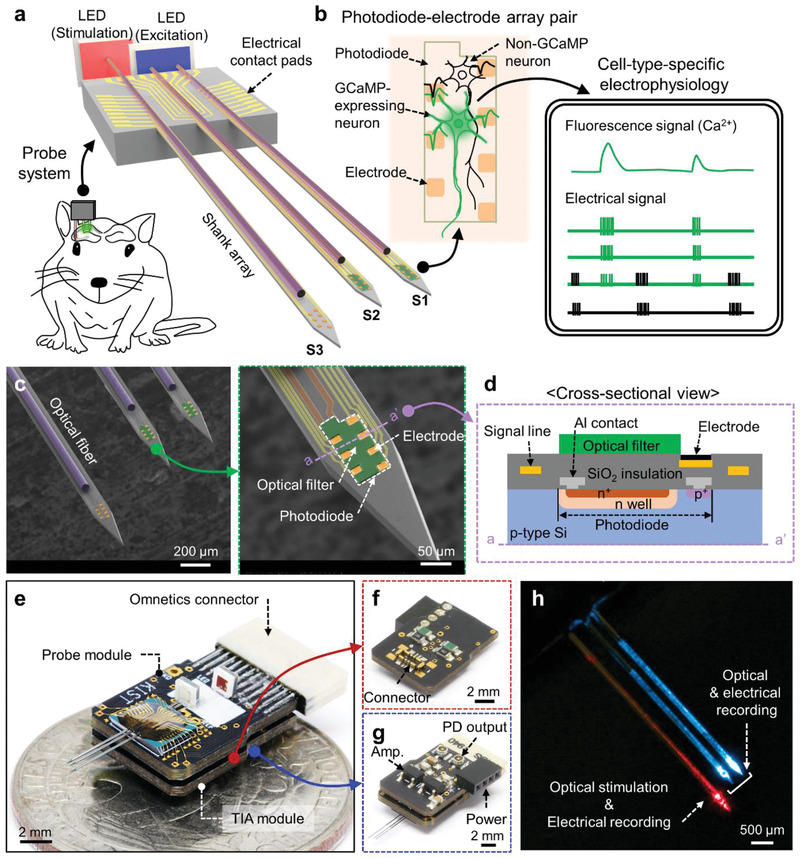
Design, fabrication, and packaging results of the multimodal fluorescence probe system. a) Schematic illustration of the multi‐shank and multi‐functional probe for the simultaneous recording of optical (S1 and S2) and electrical (S1–S3) signal with optical stimulation (S3) in neural circuits. b) Schematic illustration of cell‐type‐specific electrophysiology from GCaMP‐expressing neuron through simultaneous measurement of both optical and electrical signal. c) SEM images of the fabricated multi‐shank probe and an individual shank. The optical filter and the electrode are colored by green and orange, respectively. The Ti/Au signal lines for a photodiode and electrodes are labeled by red and yellow. d) Cross‐sectional view of the fabricated probe shank. The colors indicate the materials of each component. (Figure S3, Supporting Information). e) Mesoscale view of the packaged probe system of probe and TIA module. f) Image of the TIA module from the connector side. g) Backside view of the packaged system. h) An example operation of the system for excitation and stimulation light.

We fabricated multimodal fluorescence neural probes, miniature devices that can perform cell‐type‐specific electrophysiology from deep regions of the brain (**Figure** **1**a). The length of the shank of the probe was designed to be 6 mm so that the probe can access the entire brain region of an adult mouse. The probes were designed in single‐shank and multi‐shank configurations so that a device can be used for either a single deep brain region or larger neural circuits (Figure S1, Supporting Information). The probe structure is built using a single‐crystalline silicon substrate, which has high mechanical strength (Young's modulus = 190 GPa). Thus, the probe can be fabricated with lengths longer than tens of millimeters without compromising the structural stability of the shanks during insertion of the probe into brain tissue. While the multi‐shank probe can be designed and fabricated in any shape and configuration depending on the target application, the multi‐shank probe we utilized in this work was designed specifically for the analysis of the cell‐type‐specific connectivity in the hippocampal CA3‐CA1 circuit. More specifically, the lengths of the shanks were designed to be different so that, when the probe is inserted perpendicular to the ML‐AP plane, one shank (S3) is placed in CA3 and the other two shanks (from the right, S1 and S2) are placed in CA1. Each shank includes the electrode array for the measurement of electrical signals. Photodiode was integrated in S1 and S2 for the recording of optical signals. An optical fiber was attached on each shank to deliver excitation light (S1 and S2) and to attain precise optical stimulation (S3).

The relative location of photodiode from fiber tip for optical stimulation should be carefully designed to minimize the crosstalk because the scattering of red light from the optical fiber tip would affect the optical output of the photodiode. The baseline current of the photodiode is affected by both excitation light and base emission fluorescence in GCaMP‐expressing mouse brains, and the red light induces an additional shift on the baseline current. The baseline shift value by the red light, calculated from the recorded photodiode current of in vivo experiment, was 5.7% and 7.9% for photodiodes S1 and S2, respectively. As the distance between the photodiode and the optical fiber tip decreases, the intensity of the scattered light increases, and the level of baseline shift increases as well. To calculate the intensity of the scattered light as a function of the distance from the tip of the fiber, we conducted a Monte Carlo simulation (Figure S2, Supporting information) and confirmed that the measured baseline shift value corresponds well to the baseline shift expected from the simulation results. If the baseline of the fluorescence signal doubles, the change in the fluorescence signal compared to the baseline (Δ*F*/*F*_0_) would be reduced to half. Therefore, the distance between the optical fiber and fluorescence recording shank should be designed in consideration of both the baseline shift and the amplitude of the fluorescence signal by a red light for optical stimulation.

The key feature of the multimodal fluorescence neural probes is that it enables cell‐type‐specific electrophysiology through the simultaneous measurement of both the optical and electrical signals from a neuron (Figure 1b). We utilized microfabrication techniques to monolithically integrate all the components on the small and flat surfaces of the silicon shanks. The electrodes were placed in the vicinity of the active area of the photodiode at a very high density (50‐µm inter‐electrode distance). Seven electrodes were placed on the top of the photodiode along its boundary (Figure 1c). The pitch of the small inter‐electrode allows the accurate measurement of the activities of all the neurons that are located above the active region of the photodiode, and it enables the estimation of the location of the neuron with triangulation.^[^ ^34^ ^]^ This monolithic configuration allows the estimation of the location and activity of the specific type of cells among the various types of neuronal activities above the active area of the probe.

The photodiode was designed to have a wide active area, allowing it to collect most of the photons emitted from the soma of the neuron located on top of it. The top view of the photodiode structure is shown in the inset with a blue dashed boundary in Figure S3a (Supporting Information), and the cross‐sectional view is in Figure 1d. We used a p‐type silicon substrate with a low boron doping concentration (1 × 10^15^ cm^−3^) to form a photodiode with a large active area that had a wide and deep n‐well. The ion‐implanted n^+^ and p^+^ regions provided contact between the n‐well and the p‐type substrate, respectively. Structurally, the photodiode was embedded underneath the surface of the shank, and the electrodes were placed so that they partially overlaid the active area and the contact lines of the photodiode. A narrow‐band‐block filter was placed on the photodiode to efficiently block the excitation light and the passing of the only fluorescent emission. The optical filter was photolithographically defined so that it covered the entire photodiode area except for the electrodes. The filter was formed with a small thickness of 2.4 µm to minimize the distance between the neuron and photodiode. This monolithic configuration of the probe enabled the successful implementation of a large‐area photodetector, an electrode array, and an optical filter mounted on the small available surface of the probe shank. From these probe configurations, the thickness and width of the probe (i.e., the shank) were 40 and 113 µm, respectively. Such miniaturization of the cross‐sectional area is expected to reduce the damage to the tissue and improve the quality of the recorded neuronal signals.

The bimodal and multi‐shank fluorescence probe that was fabricated was attached on a printed circuit board (PCB) with an optical fiber and surface‐mount LEDs for both excitation and light stimulation (Figure 1e). We also designed the readout circuit of the fluorescence signals with a transimpedance amplifier (TIA) using a commercial operational amplifier (Op‐amp) chip that was attached to a separate PCB (Figure 1f,g). The TIA circuit converts the photocurrent from the probe to voltage with a gain of 10^7^. In the proposed system, the electrical signals were transferred to the pre‐amplifier of the recording system (RHD Recording Headstages, Intan Technologies) through the omnetics connector, and the fluorescence signals were transferred to the TIA PCB through connectors located on both PCBs. The circuit diagram of the entire system is provided in Figure S5 (Supporting Information). During the operation of the probe, the lights for both the excitation of the genetically encoded calcium indicators (GECIs, e.g., GCaMP6) and the stimulation of the opsins (e.g., ChrimsonR) are provided from the tips of the integrated optical fibers (Figure 1h). The modular, multi‐stack PCB packaging enables the integration of the multi‐shank array in a miniature system.

### 2.2 Characterizations of Fluorescence Neural Probe

**Figure 2:**
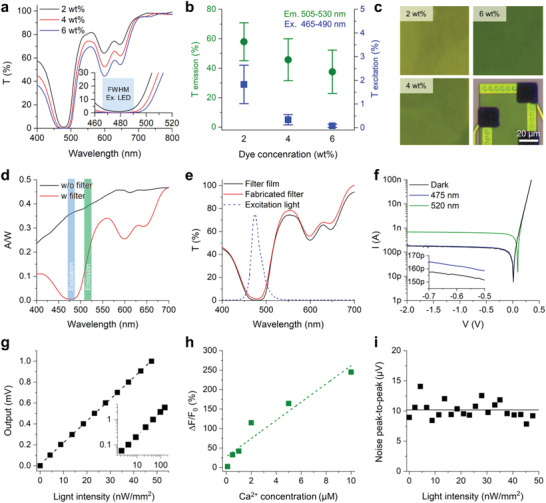
Electrical and optical characterizations of the fluorescence neural probe. a) A transmittance spectrum of a narrow‐band‐block filter with 2 (black), 4 (red), and 6 (blue) wt% color dye in SU8 2002. The inset graph shows a zoom‐in plot over the central wavelength range of LED for GCaMP excitation. b) Transmittance values of varied dye concentrations of 2, 4, and 6 wt% in the emission and excitation light range. Data represented as mean ± SD. c) Images of the 2, 4, and 4 wt% filter coated slide glass and the integrated filter on the photodiode. d) A responsivity plot from the probe with (red) and without (black) a narrow‐band‐block filter integrated on the photodiode. e) Comparison of the transmittance spectrum between the filter integrated on the photodiode and the slide‐glass. The wavelength spectrum of excitation light from an integrated LED is indicated by the blue dashed line. f) An *I*–*V* curve of the fabricated photodiode in a dark state and under 1 µW light of 475 and 520 nm light and in a bias voltage range from 0.5 to −2.0 V. g) An output plot of the packaged system from an external green LED with light intensity from 2 to 150 nW mm^−2^. h) Measured value of fluorescence intensity in calcium indicator with a varied calcium ion concentration. i) A noise peak‐to‐peak plot of the system under the green LED light ranges from 2 to 48 nW mm^−2^ light intensity.

We characterized the optical and electrical performances of the individual components of the integrated system to evaluate the system's recording capability. To record the fluorescence signal from cells, we need to block the excitation light because it has a higher light intensity than the fluorescence signal. Thus, we need a high‐performance optical filter that can reject the wavelength of the excitation light. First, we characterized the transmittance of the 2.4‐µm‐thick filter with various concentrations (2, 4, and 6 wt%) of dye mixed with SU‐8 (**Figure** **2**a,b). Under the illumination of white light, the colors of the fabricated filters darkened from light green to dark green depending on the dye concentration of 2–6 wt% (Figure 2c). In the wavelength range of 460–520 nm, the light rejection of the filter coincided with the peak wavelength of the blue LED (from 465 to 488 nm) that was used as the excitation light source (inset of Figure 2a). The emission from GCaMP6 GECI is known to have a peak wavelength between 510 and 520 nm. The transmittance ratio of the emission light to the excitation light increased as the concentration of the dye increased. Although higher rejection of the excitation light was achieved with higher concentrations of the dye, we used the filter that had a dye concentration of 4 wt% dye so that as much emission light as possible could be received while the crosstalk from the excitation light was minimized.

Then, we characterized the optoelectrical performance of the photodiode that was integrated with the filter. We measured the responsivity (A W^−1^) curves of a photodiode measured between the wavelengths of 400 and 700 nm (Figure 2d). It was apparent that the 4 wt% filter effectively blocked the excitation light and passed the GCaMP6 emission light. The responsivity at *λ* = 475 nm decreased from 0.36 without the filter to 0.48 × 10^–2^ with the filter, while the responsivity at *λ* = 520 nm decreased only slightly, i.e., from 0.40 to 0.22. We calculated the transmittance spectrum of the filter integrated on the photodiode from the change of responsivity values and confirmed that the calculated spectrum corresponded well to values measured using the spin‐coated filter on glass (Figure 2e). Both results confirmed that the fabricated filter successfully blocked the excitation light with an attenuation of −18.86 dB (98.7%) at 475 nm. Then, we swept the bias voltage across the anode–cathode terminals and measured the *I*–*V* curves of the filtered photodiodes in the dark, under 475‐nm light, and under 520‐nm light (Figure 2f and Figure S6a, Supporting Information). No significant reverse‐bias leakage current was measured, and the breakdown voltage of the photodiode was as high as −56 V. We confirmed that the performance of the probe with an integrated photodiode could achieve the measurement of the selective wavelength of the green fluorescent protein (GFP) emission with a rejection of the excitation wavelength.

The linearity, dynamic range, and noise performance of the multimodal fluorescence probe system, which are important parameters for fluorescence sensors, were also characterized. To evaluate the linearity of the system, we measured the output voltage from the packaged probe system by applying 520‐nm LED light (green, GCaMP emission wavelength) at various light intensities from 2 to 150 nW mm^−2^, which covered the conventional fluorescence intensity of GCaMP, and the system showed excellent input‐to‐output linearity (*R* ^2^ = 0.999; Figure 2g). Then, to confirm the dynamic range of the photodiode, we measured the fluorescent emission intensity in a range of intracellular calcium levels in neurons using Oregon Green 488 BAPTA‐2 as the calcium indicator (Figure 2h). The typical intracellular resting calcium concentration is known to be 50–100 × 10^−9^ m. During the electrical activity of a neuron, the calcium level can increase by as much as 100 times the baseline concentration.^[^ ^35^, ^36^ ^]^ The minimum detectable fluorescence signal was 2.4% at 0.1 × 10^−6^ m, and the fluorescence signal increased linearly with the calcium concentration up to 245.1% at 10 × 10^−6^ m. The coefficient of determination (*R* ^2^) of the linear fitted data from the measured fluorescence from 0.1 to 10 × 10^−6^ m was determined to be 0.907. Then, we measured the noise level of the system at various GCaMP emission light intensities (Figure 2i). The peak‐to‐peak amplitude of the noise was below 15 µV, which corresponds to 1.5 pA of photocurrent. Considering that the photodiode's dark current was 155 pA and the photocurrent due to the baseline fluorescence (Figure 2h) was ≈19.7 nA, the noise amplitude of the system was negligible. These data indicated that the noise and recording performance of the probe system after the integration of the transimpedance amplifier module confirmed the recording of the ex vivo and in vivo GFP signals.

To estimate the photometric performance of the system, we measured the acceptance angle of the photodiode and simulated the spatial distribution of the excitation light intensity and capturing volume of the photodiode, both using Monte Carlo simulations. The measured acceptance profile of the photodiode showed that the photodiode captures much of light with an incidence angle between 45° and 135°, as much as more than 50% of that with an incidence angle of 90°. In the proposed system, the photodiode and the optical fiber are orthogonally placed, and the excitation light illuminates the volume directly above the photodiode (Figure S7b, Supporting Information). From the simulation result of the acceptance profile of the photodiode, we confirmed that that the photodiode captures fluorescence almost exclusively from the volume right above its surface (Figure S7c, Supporting Information). The capturing volume of the photodiode was determined by the product of the fluence profile of fluorescence and the acceptance profile of the photodiode (Figure S7d, Supporting Information). The capturing volume of the photodiode, defined as the volume from which 90% of the total captured fluorescence is generated, was calculated to be 1.28 × 10^6^ µm^3^ (Figure S7d, Supporting Information).

Also, we assessed the electrical characteristics of the Pt black electrodes. We used electrochemical impedance spectroscopy (EIS) and measured the impedance of four electrodes from two sample probes (Figure S6b, Supporting Information). The magnitude and the phase of the impedance at 1 kHz were 16.1 ± 0.1 kΩ and 35.7 ± 1.7° (mean ± SD), respectively. These values are comparable to the values reported in previous studies that used Pt‐black electrodes,^[^ ^37^ ^]^ so they are low enough for in vivo extracellular electrophysiology.

To confirm the long‐term stability of the probe system, we performed an accelerated aging test using a 67 °C 1× phosphate‐buffered saline (PBS) solution for 5 d (Figure S8, Supporting Information). The elevated temperature provides an acceleration factor of 8 compared to 37 °C.^[^ ^38^ ^]^ There was little change in the impedance and dark current of the photodiode measured from the probes in the accelerated aging conditions for 5 d (Figure S8, Supporting information). Based on these results, we suggest that the fluorescent probe would maintain its optical and electrical signal recording functionalities for 40 d in vivo.

### 2.3 Comparison of Fluorescence Neural Probe and Fluorescence Microscopy through Ex Vivo Recording of Calcium Changes

**Figure 3:**
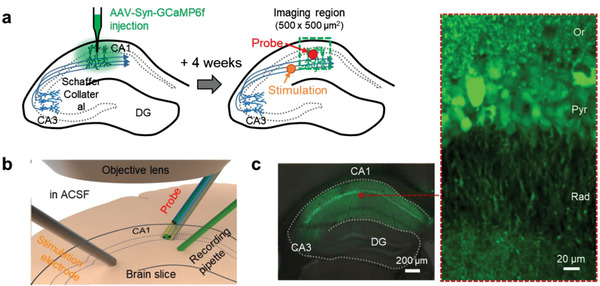
a) Schematic of the experimental method of the ex vivo recording from AAV‐Syn‐GCaMP6f expressed mice in the CA1 region. b) Schematic position of the probe shank, a glass recording pipette, a stimulation electrode, and an objective lens in incubation chamber for the recording of calcium activities from the brain slice. c) Fluorescence images in the hippocampus region from fixed brain slices of Syn‐GCaMP6f expressed mice.

**Figure 4:**
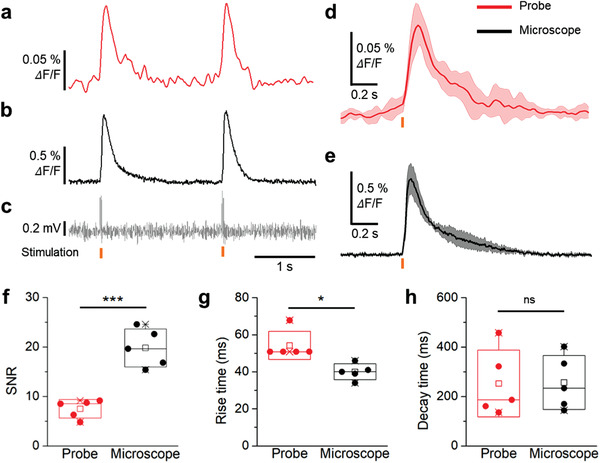
Comparison between the fluorescence neural probe and a conventional fluorescence microscope through the recording of calcium changes from a brain slice. Recording results of calcium activities from the a) fluorescence probe system and b) fluorescence microscope. c) Recorded electrical pulses during the current stimulation from the stimulation electrode. The timing point of electrical stimulation is indicated by yellow bars. d,e) Accumulated calcium activities from both systems, simultaneously. f) SNR, g) rising time, and h) decay time plots calculated from the measured calcium signal (*p*‐value: 6.61 × 10^−4^ (SNR), 1.06 × 10^−2^ (rising time), 0.96 (decay time), *n* = 5 from two mice, two‐tailed unpaired *t*‐test). Data represented as mean ± SD. *P < 0.05; **P < 0.01; ***P < 0.001; ****P < 0.0001; NS: no significant difference.

To validate the capability of the proposed probe system, we recorded calcium signals in the hippocampal CA1 region of a brain slice using both the fluorescence neural probe and conventional fluorescence microscopy (**Figure** **3**a). Hippocampal CA1 of mice brain was transfected with adeno‐associated virus (AAV) expressing the genetically encoded calcium indicator (GECI) Syn‐GCaMP6f. Four weeks after the injection, we sliced the mouse brain and obtained a slice with strong GCaMP6f expression for the imaging. The probe shank, a glass recording pipette, a stimulation electrode, and an objective lens were optimally positioned in the small chamber to record the calcium activities from the brain slice (Figure 3b). We observed GCaMP expression in the entire CA1 region (Figure 3c). With the sufficient GCaMP expression confirmed, we simultaneously recorded the evoked calcium signals that were induced by the electrical stimulation through both the probe and the microscope (**Figure** **4**a,b). Along with the calcium signal recording, the stimulation signal also was recorded near the probe using the recording pipette (Figure 4c). The traces of calcium signals (*n* = 5 trials in two brain slices) recorded from the probe and the microscopes were compared for the statistical analysis (Figure 4d,e). Due to the difference in the neuronal population, the recording area, and the baseline rejection from which the fluorescence signal was collected, the amplitude of the change in the calcium signal measured using the conventional fluorescence microscope was about nine times greater than that measured using the probe. Even so, the shapes of the fluorescence signals were similar (Figure 4a–e and Figure S9, Supporting Information). The signal‐to‐noise ratio (SNR) of the signals recorded using the microscope also was higher than that of the signal recorded using the probe system (Figure 4f). Although the noise level was slightly higher, the probe exhibited sufficiently high SNR (7.5 ± 1.7, mean ± SD) to record the calcium activity. The similarity of the signals measured using the two systems was further confirmed by comparing the rise and the decay times of the waveforms (Figure 4g,h). Note that the slightly longer rise time of the probe signal (longer by 14.2 ms) is due to the lower sampling rate (60 Hz vs 1 kHz of the microscope signal) of the signal measured by the probe. The time constants of the recorded signals suggested that both systems have the capability to accurately depict the kinetics of GCaMP6f signals.^[^ ^39^ ^]^ Overall, the results confirmed that the fluorescence neural probe system successfully recorded accurate GECI signals of neuronal calcium dynamics.

### 2.4 In Vivo Cell‐Type‐Specific Electrophysiology for Spontaneous Neural Activity Recording

**Figure 5:**
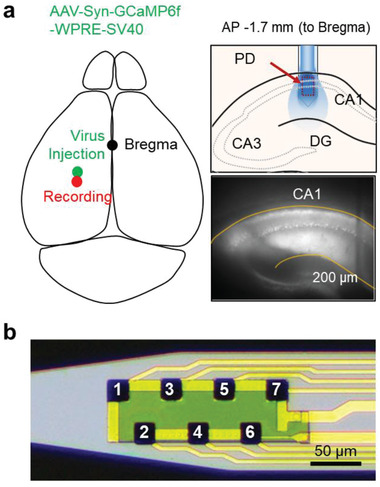
a) Schematic illustration of the experimental method for in vivo recording using the single‐shank probe. b) The optical image and channel information of the single‐shank probe.

**Figure 6:**
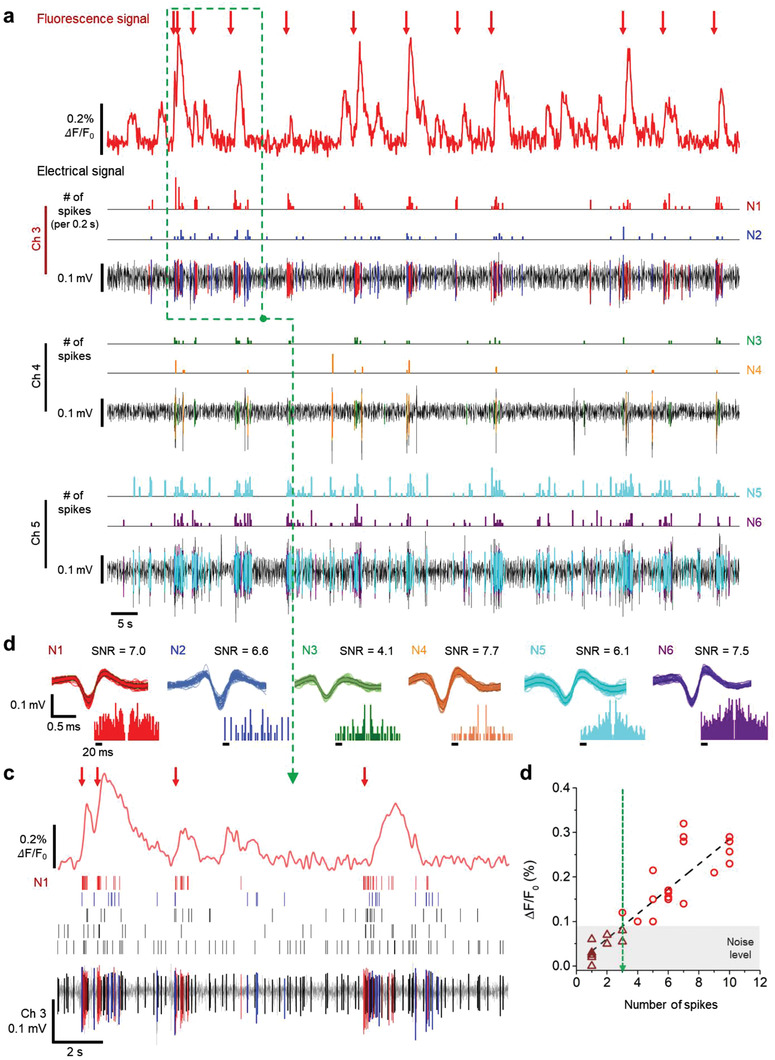
Simultaneous in vivo recording of fluorescence and electrical signal for spontaneous neural activity in the CA1 region. a) An image of the recording area at the tip of the shank. The location of the identified channels correlated with calcium changes is indicated by a red circle. b) Waveform, autocorrelogram, and SNR of sorted neural spikes that seem to contribute to calcium change. c) Zoom‐in plot of the green box with a dashed line of (a), which shows the recorded electrical and fluorescence signals from channel 3 and the photodiode, respectively. d) Calcium change as a function of the number of N1 spikes in a period of 200 ms. The green dashed line represents the minimum number of electrical spikes associated with detectable calcium changes.

After validating the capability of the fluorescence neural probe system to detect calcium signals ex vivo, we confirmed the system's capability of simultaneous optical and electrical recordings in an in vivo environment for the cell‐type‐specific electrophysiology. We implanted a single‐shank probe into the hippocampal region of mice injected with AAV1‐hSyn‐GCaMP6f (**Figure** **5**). First, the probe was inserted into the brain of a mouse until its tip reached the pyramidal layer of the hippocampal CA1 region. Then, the probe recorded spontaneous neural activity by measuring the calcium and electrical signals simultaneously (**Figure** **6**a and Figure S10, Supporting Information). The change in the calcium signal coincided well with the putative single‐unit activities recorded in channel 3 (Figures 5b and 6a). While many spikes seemed to contribute to the recorded calcium activity (Figure 6b), the N1 neuron, recorded from channel 3, had the highest correlation to the change in the calcium signal. The high correlation suggests that this spike could have originated from the same neuron from which the calcium signal was being recorded (Figure 6c). The time points when neuron N1 fired consecutively are indicated by the red arrows on the calcium plot (Figure 6a,c). Further analysis of the relationship between the amplitude of the calcium signal and the N1 neuron's spiking rate showed the existence of a linear relationship (*R* ^2^ = 0.79; Figure 6d). The linearity between these two parameters was defined similarly in previous studies which used conventional microscopes and a single‐cell patch electrode.^[^ ^39^, ^40^, ^41^ ^]^ From noise analysis, we concluded that the photodiode could reliably detect the change in the calcium signal originating from more than four consecutive spikes within a 200‐ms period (Figure 6d, a gray area and green dashed line). In addition, we recorded evoked neural activity during a pharmacologically induced seizure from the hippocampal CA1 region to show the correlation between electrical fluorescence signals, and we measured high fluorescence signal that was simultaneously generated with high‐frequency neural spikes (Figure S11, Supporting Information). These in vivo results indicated that our system could record and analyze in vivo calcium and electrical signals simultaneously in the deep brain region.

**Figure 7:**
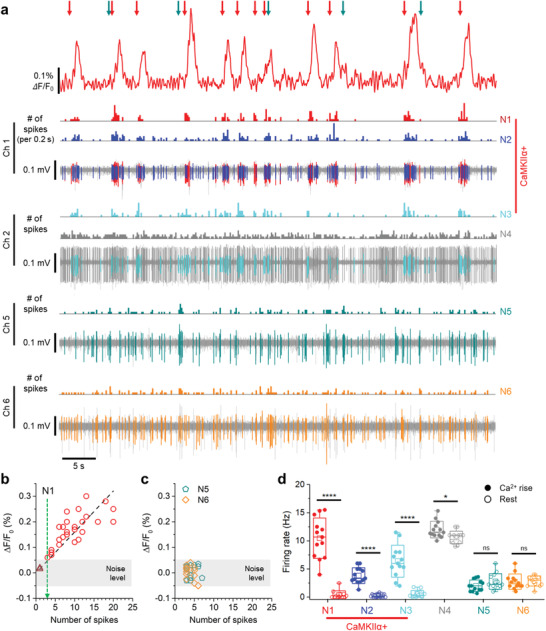
In vivo cell‐type‐specific electrophysiology at CA1 from CaMKII*α*‐GCaMP6f expressing mice. a) Representative plot of the simultaneous recording of electrical and fluorescence signals with the cell‐type identification of N1–N6 spikes using the fluorescence probe. Spikes of the excitatory neuronal activity contributes to calcium is indicated with a red arrow, and the unspecific neuronal activity that does not contribute is marked with a dark cyan arrow. b) Calcium change as a function of the number of spikes from N1 neuron in a 200 ms period. The green dashed line represents the minimum number of electrical spikes associated with detectable calcium changes. c) Calcium change as a function of the number of spikes from N5 and N6 neuron in a 200 ms period (The data points were analyzed and plotted only in periods with three or more spikes). d) The average firing rate of the N1–N6 neurons during calcium rising and resting state. The data were analyzed using two‐tailed unpaired *t*‐test (*p*‐value: 2.02 × 10^−7^ (N1, Ca^2+^ rise‐rest), 6.47 × 10^−7^ (N2), 6.67 × 10^−6^ (N3), 1.38 × 10^−2^ (N4), 0.12 (N5), 0.84 (N6), *n* = 13 for each neurons). Data represented as mean ± SD. *P < 0.05; **P < 0.01; ***P < 0.001; ****P < 0.0001; NS: no significant difference.

After the system's capability of simultaneously recording the electrical and the optical signals was confirmed, we were able to demonstrate the ability of the system to conduct the cell‐type‐specific electrophysiology. We used mice injected with AAV1‐CaMKII*α*‐GCaMP6f virus to analyze the activities of the CaMKII*α*‐positive (CaMKII*α*+) neural subpopulation in the experiment. The electrical signals, including both spontaneous and burst activities, were recorded in channels 1, 2, 5, and 6, and the signals were sorted to investigate their correlations with the promotor‐selective fluorescence signals (**Figure** **7** and Figure S12, Supporting Information). The change in the calcium signal coincided with the putative single units of N1, N2, and N3 neurons recorded from channels 1 and 2 (Figure 7). Unlike the N1, N2, and N3 electrical recordings, the observed neural spikes of the N5 and N6 neurons did not accompany any significant changes in the fluorescence signal. Similarly, the calcium signal was not affected by the neural spikes of N4, although its firing rate was high enough to induce changes in the calcium. From these results, we confirmed that only the activities of the CaMKII*α*+ neurons contributed to the fluorescence signal. These results strongly indicated that the multimodal fluorescence probe system enabled the measurement of the cell‐type‐specific electrophysiology at a sub‐millisecond temporal resolution.

### 2.5 In Vivo Cell‐Type‐Specific Electrophysiology in Multiple Regions of Hippocampal Circuit

**Figure 8:**
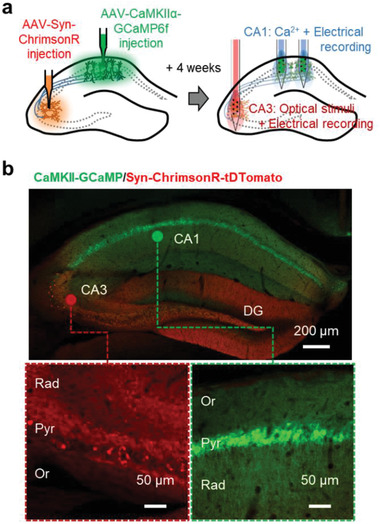
a) Schematic illustration of the experimental methods for the hippocampal circuit study. b) Fluorescence images resulting from brain slices of a mouse in which Syn‐ChromsonR and CaMKII*α*‐GCaMP6 were expressed in the CA3 and CA1 regions, respectively.

After confirming that the combination of a photodiode and an electrode array enables the cell‐type‐specific electrophysiology within a small volume of the brain, we demonstrated that the fluorescence probe could be scaled up into a multi‐shank configuration to conduct the cell‐type‐specific electrophysiology in multiple regions across the neural circuit. We designed a multi‐shank probe that consists of three shanks with different lengths, optimized for the investigation of a well‐known hippocampal pathway (**Figure** **8**a and Figure S13, Supporting Information), i.e., the Schaffer collateral.^[^ ^42^ ^]^ In this experiment, we used periodic optogenetic stimulation to activate cells of a specific type in the CA3 region so that activities of multiple neurons in the CA1 region can be elicited through the axonal projections during the stimulation and, in turn, the increment of the activities of cells of a specific type can be clearly demonstrated. We chose ChrimsonR as the excitatory opsin for CA3 neurons because it can be excited using light with wavelength far enough from that of GCaMP and thus used with minimal crosstalk. ChrimsonR was delivered into the CA3 region with a viral vector with a Synapsin promoter. At the same time, AAV carrying CaMKII*α*‐GCaMP6f for the selective transfection to excitatory neurons was injected into the CA1 region (Figure 8a). Four weeks after the injection, each protein was expressed strongly in each region without significant overlap (Figure 8b).

**Figure 9:**
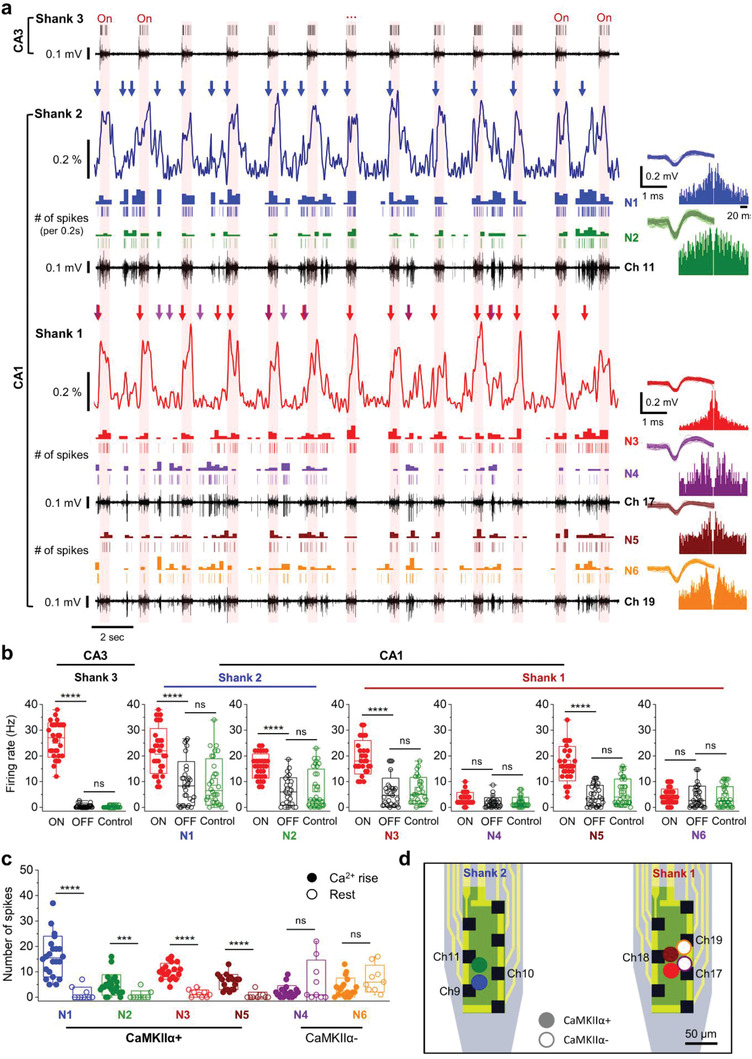
Investigation of in vivo hippocampal circuit through the cell‐type‐specific electrophysiology in multiple regions. a) Representative plots of the recorded electrical and fluorescence signals from the multi‐shank probe during light stimulation (100 µW, 0.5 Hz, 25% duty cycles; red background indicates the periods of optical stimuli). The waveform and the autocorrelogram of the identified neurons from the plotted channels are shown on the right. b) Comparison of the firing rate of onset (ON) and offset (OFF) cycle during optical stimulation, and spontaneous activity before stimulation (Control). The data were analyzed statistically using the two‐tailed, unpaired *t*‐test (*p*‐value: 2.03 × 10^−19^ (S3, ON‐OFF), 0.634 (S3, OFF‐Control), 4.36 × 10^−7^ (N1, ON‐OFF), 0.773 (N1, OFF‐Control), 3.21 × 10^−10^ (N2, ON‐OFF), 0.491 (N2, OFF‐Control), 1.32 × 10^−12^ (N3, ON‐OFF), 0.627 (N3, OFF‐Control), 0.362 (N4, ON‐OFF), 0.342 (N4, OFF‐Control), 5.86 × 10^−10^ (N5, ON‐OFF), 0.234 (N5, OFF‐Control), 0.848 (N6, ON‐OFF), 0.958 (N6, OFF‐Control), *n* = 30 for each of the neurons). c) The average firing rate of rising and resting state of calcium signals from the CaMKII*α*+ and CaMKII*α*‐ neurons (*p*‐value: 2.47 × 10^−8^ (N1, Ca^2+^ Rise‐Rest), 5.17 × 10^−4^ (N2), 3.53 × 10^−10^ (N3), 0.303 (N4), 9.48 × 10^−7^ (N5), 9.56 × 10^−2^ (N6), *n* = 22 and 18, two‐tailed unpaired *t*‐test). Data represented as mean ± SD. d) Schematic illustration of the estimated location of CaMKII*α*+ and CaMKII*α*− neurons with triangulation. *P < 0.05; **P < 0.01; ***P < 0.001; ****P < 0.0001; NS: no significant difference.

First, we confirmed that the multi‐shank probe can determine cell‐type‐specific activities in multiple regions across the neural circuit through the process of recording both the electrical and the fluorescence signals (**Figure** **9**a and Figure S14a, Supporting Information). From the electrodes on Shanks 1 and 2, both spontaneous and light‐induced burst activities were observed. Both the spontaneous and the light‐induced activities contributed to the fluorescence signal, which was similar to those observed with the single‐shank probe, as presented in Figure 7. In these experimental results, we observed clear differences among the contributions of the individual cell's burst firing to the fluorescence signal. The time points when neurons of N1 (recorded from Shank 2) and N3 (from Shank 1) consecutively fired more than four times within a 200‐ms period are indicated by the blue and red arrows, respectively, above the calcium signal traces. Significant changes in calcium signals from these cells were recorded as spikes in the photocurrent measured by each photodiode. However, burst firings of N4 (from Shank 1), indicated by the purple arrows, did not accompany any significant change in the recorded fluorescence signal. These results showed that the sorted spikes could be classified into two groups by the correlation between electrical and fluorescence signals. We classified N1, N2, N3 and N5 neurons as CaMKII*α*+ neurons due to the strong correlation between electrical and fluorescence signals, while we classified N4 and N6 as CaMKII*α*‐ neurons because electrical and fluorescence signals didn't show any correlations.

To confirm the type of cells that make up the neural circuit, we statistically analyzed the correlation between firing rate and fluorescence signal. First, we calculated the firing rates of each identified neuron with and without optical stimulation. The average firing rate of the neuron recorded from Shank 3 (in CA3) showed a statistically significant increase during the light stimulation (Figure 9b). Also, the firings of the neurons of N1, N2 (from Shank 1), N3, and N5 (from Shank 2), all of which are located in CA1, were well synchronized with the optical stimulation, which means that these neurons are functionally connected to CA3 region. On the other hand, N4 and N6 neurons exhibited only spontaneous burst activities that did not correlate well with the optical stimulation in CA3. Thus, we can classify the neurons in CA1 region into two groups according to the responses by optical stimulation in CA3. Then, we can classify which groups are CaMKII*α*+ neurons by the recorded fluorescence signal. There were increase of spikes during the “rise time” in the calcium signal in neurons of N1, N2, N3, and N5 (Figure 9c), while N4 and N6 neurons did not show any increase of firing rate during the “rise time” in the calcium signal. These results indicate that the groups which showed increased neural activities during optical stimulation are the CaMKII*α*+ neuron group. We could also estimate the location of CaMKII*α*+ and CaMKII*α*− cells by the analysis of signals from electrode array with triangulation (Figure 9d and Figure S14b–d, Supporting Information).

As these findings represent, the multi‐shank fluorescence probe system enables to find out the type of cells which make up the neural circuits among specific sub‐populations of neurons and thereby allows in‐depth study of cell‐type‐specific functional connectivity inside the neural circuit. Thanks to the scalability of the monolithically integrated platform, the cell‐type‐specific neural activities can be monitored from multiple regions across the neural circuit, further expanding the capability of the system. Therefore, we expect the proposed system to be used in a variety of experiments for the in‐depth analysis of neural circuits at high temporal resolution with identification of cell‐type‐specific functional connectivity in vivo.

## 3 Discussion

We present a multimodal fluorescence neural probe that can perform cell‐type‐specific electrophysiology in multiple regions across neural circuits in a deep brain, at a cellular spatial resolution and a sub‐millisecond temporal resolution. For example, the cell‐type‐specific electrophysiology enables the in‐depth analysis of the role of a specific type of neurons within the neural circuit. By performing this analysis at various regions in a neural circuit simultaneously, we can analyze, at the same time, the role of a specific subset of neurons in the neural circuit and the connectivity of those neurons to other neurons which are not included in the subset. Therefore, the proposed system would enable studies of complex neural circuits composed of various types of neurons that have different roles.

We confirmed that the proposed multimodal fluorescence neural probe could record cell‐type‐specific electrical signals from the spontaneously firing CaMKII*α*+ neurons. The probe in the multi‐shank configuration could have been used without an optical stimulation to record spontaneous activities of neurons with cell‐type identification. However, for the in vivo experiment in multiple regions of the hippocampal circuit, we used a multi‐shank probe with optical stimulation capability. Considering the mainly excitatory connections in the CA3–CA1 pathway, the type of the cells whose activities were evoked could have been identified as excitatory as well. In this experiment, we used optical stimulation to evoke activities of CaMKII*α*+ neurons and thus clearly demonstrate the cell‐type‐specific electrophysiology in multiple regions. It would be possible to record cell‐type‐specific electrophysiological signals from different sub‐population of neurons such as dopaminergic, GABAergic, glutamatergic neurons from multiple regions, as long as they are connected to the optically stimulated neurons.

We used calcium signals for the analysis of the cell‐type‐specific dynamics of neurons and analyzed the correlation between the electrical signal and the calcium signal. Most of the recorded calcium spikes were in good correspondence with the spikes observed in the electrical signals, i.e., 91.7 ± 4.9% from six mice. However, we observed a few spikes in the calcium signal that did not correspond to any of the spikes in the electrical signal (Figure S15, Supporting Information, indicated with green asterisks). The non‐matched calcium spikes might be generated by neurons located near the photodiode but far from electrodes. We can increase the correspondence by optimization of electrode locations or increasing the number of electrodes.

With some modification of the configuration of the probe and the signal recording system, the next versions of the device may enable more advanced animal experiment paradigms, such as large‐scale brain recording or experiments using freely behaving animals. The monolithic configuration of the integrated probe facilitates the scaling of the device for large‐scale brain monitoring applications. With a greater number of photodiodes and electrodes integrated on each shank and with more shanks, cell‐type‐specific electrophysiology from much wider brain region(s) can be conducted using a single probe array in a brain. Alternatively, by integrating all of the components of the recording circuit with a wireless transceiver, a microcontroller, and a battery, the packaged probe system can be used in a wireless configuration, which would allow the analyses of the brain activities in awake, freely moving animals. In addition, the chronic spike recording performance of the electrodes needs to be assessed for applying the system to long‐term chronic applications. Our previous work successfully showed the long‐term recording capability of electrodes in freely moving animals for one month.^[^ ^43^ ^]^ As the electrodes presented in the previous work are almost identical to the fluorescence probe presented in this work in terms of dimensions, materials, and fabrication process, it is safe to conclude that we can utilize the fluorescence probe in long‐term chronic applications as well.

The closely packed configuration of the photodiode and electrode array can also be used for the simultaneous measurement of a variety of optical signals, which may reflect a number of different neuronal and physiological states with electrical signals. For example, with genetically encoded fluorescent indicators,^[^ ^20^, ^44^, ^45^, ^46^, ^47^, ^48^ ^]^ the probe can optically measure vesicular release,^[^ ^46^ ^]^ neurotransmitter level,^[^ ^47^ ^]^ and transmembrane voltage.^[^ ^48^ ^]^ The combination of the color filter and the wavelength of the LED in the probe system can be modified so that fluorescence signals of a different color can be recorded efficiently. With the combination of multiple genetically encoded indicators (GEIs) with different emission wavelengths, an integrated neural probe system with multiple filters and LEDs would enable the analysis of the roles of several different types of neurons in an intricate neural circuit.

## 4 Experimental Section

### Fabrication of the Fluorescence Neural Probe

The fabrication procedure of the fluorescence neural probe is illustrated in Figure S3 (Supporting Information). A 4‐in. silicon‐on‐insulator (SOI) wafer that had device, buried oxide (BOX), and handle layer thicknesses of 40, 0.7, and 450 µm, respectively, was used to fabricate the neural probe. The device layer was p‐type silicon with a resistivity of ≈14 Ω cm. For each step in the photolithography process, a soft photoresist mask formed by a standard contact photolithography process was used, and the masks at the end of each process were removed. First, a thick (700‐nm) SiO_2_ layer that served as an implantation mask was grown by wet oxidation on a pre‐cleaned SOI wafer. Next, the SiO_2_ layer was etched by the reactive ion etching (RIE) process to form a hard mask for n‐well doping. The implantation of phosphorus ions (200 keV, 2 × 10^13^ cm^–2^) was performed, and this was followed by a drive‐in diffusion process for 20 min at 1000 °C. Photoresist soft masks were used for the implantations of n^+^ and p^+^. Arsenic (60 keV, 3 × 10^15^ cm^–2^) and BF_2_^+^ (40 keV, 3 × 10^15^ cm^–2^) implantations were conducted to form the n^+^ and p^+^ contact, respectively. After each implantation step, the annealing process was performed at 900 °C for 20 min.

After the formation of the photodiode, first, a 200‐nm‐thick SiO_2_ insulation layer was deposited using plasma‐enhanced chemical vapor deposition (PECVD). Then, the SiO_2_ layer was patterned by a wet etching process using buffered oxide etchant (BOE) for the formation of the n^+^ and p^+^ contacts (Figure S3a, Supporting Information). A 250 nm‐thick Al layer was deposited by sputtering and patterned by an inductive coupling plasma (ICP) etching process for signal lines of the photodiodes. Then, a second layer of SiO_2_ that was 300 nm thick was deposited by PECVD to protect the Al layer, and it was patterned by RIE to form the vias for access to the Al layer for electrical contacts (Figure S3b, Supporting Information). A Ti/Au metal layer was deposited on top of the second SiO_2_ layer. This was done by sputtering with a thickness of 20/300 nm, and the metal layer was etched using ICP etching to form electrodes and signal lines. A third 450‐nm‐thick SiO_2_ insulation layer was deposited by PECVD, and it was patterned by RIE to open electrodes and contact pads (Figure S3c, Supporting Information). Then, a 10/150‐nm‐thick Ti/Pt layer was deposited by sputtering and patterned using the lift‐off process to provide an adhesion layer between the Au electrode and the electroplated Pt black layer (Figure S3d, Supporting Information).

In the proposed photodiode integrated on the neural probe, a polymer (SU‐8 2002, Kayaku Advanced Materials, Westborough, MA) was used as the base material of the filter layer. A narrow‐band absorbing color dye (ABS 473) was selected for its good solubility in cyclopentanone, the solvent for the SU‐8. A colored SU‐8 was prepared by mixing the dye with SU‐8 at a weight ratio (color dye:SU8) of 4% for the excellent rejection ratio of the excitation light. The solution was stirred at 100 rpm for 24 h in a dark environment. Then, the colored SU‐8 was spin‐coated and patterned on the photodiode region using a standard photolithography procedure (Figure S3e, Supporting Information). After the formation of the filter layer, the probes were released using the two‐step, deep reactive ion etching (DRIE) process with one step being performed on the front of the SOI wafer and then another step performed on the backside of the SOI wafer (Figure S3f,g, Supporting Information). Then, the remaining BOX layer was removed completely from the backside using an RIE process to release the probe structures (Figure S3g, Supporting Information).

### Packaging of the Fluorescence Neural Probe

A custom‐designed PCB (width, 9.2 mm; length, 9.2 mm; height, 1 mm) was used as the packaging for the fluorescence neural probe. First, a fast‐drying epoxy was used to attach the release probe to the PCB. The exposed Au pads of the probe were connected via gold wires to the pads on the PCB using a wedge bonder (4526, Kulicke & Soffa Industries, Inc., Singapore). Then, an Omnetics connector (Nano‐NPD‐24, Omnetics Connector Corporation, Minneapolis, MN) and a board‐to‐board connector (SlimStack, Molex, Lisle, IL) were soldered onto the PCB to provide electrical connections between the probe and the signal recording modules. For the delivery of the excitation light, two optical fibers with 50‐/60‐µm core/cladding diameters were aligned precisely under a microscope on the S1 and S2 of the probe, using a 6‐axis micro‐manipulator. In addition, an optical fiber that was 3 mm longer than the excitation fibers was positioned accurately on the S3 for optical stimulation. The distance between the tip of the fiber and the edge of the photodiode was adjusted to be ≈80 µm. After positioning the optical fibers, they were glued on the probe body using UV‐curable epoxy (NOA 148, Norland Products, Inc., Cranbury, NJ). One LED was used for excitation light, and another LED was used for optical stimulation. The LED for excitation light was combined with the optical fibers integrated in S1 and S2, and the LED for optical stimulation was applied to the optical fiber placed in S3. After placing LEDs (XQ‐E BLUE for excitation and XQ‐E RED for optical stimulation, Cree Inc., Dunham, NC) on the opposite end of the optical fiber, the UV‐curable epoxy, which had a refractive index that matched well with that of the fiber core, was applied to the gap between the end of the optical fiber and the emitting surface of the LED chip. Then, a thermally curable black epoxy (EPO‐TEK 320, Epoxy Technology, Inc., Billerica, MA) was applied over the junction between the LED and the fiber and cured to block the leakage of light (Figure S4, Supporting Information).

A separate transimpedance amplifier (TIA) module was prepared for the conversion of the generated photocurrent to a voltage output with sufficient amplification. A low‐noise, FET‐input operational amplifier in an SOT‐23 package (OPA657, Texas Instruments, Dallas, TX) was selected to satisfy the low noise and high gain requirements while keeping a small profile. The components of the TIA module were soldered on a custom‐designed PCB (9.2 × 12.5 mm^2^). The gain of the amplifier was set to 1 × 10^7^ V A^−1^.

Finally, the probe module was connected to both the TIA module and a commercial neural signal amplifier module (RHD2316, Intan Technologies, CA). The low‐profile board‐to‐board connector with only a 0.6‐mm coupling height provided the mechanical and the electrical connections between the probe module and the TIA module. An Omnetics connector (8/24‐pin connector for single‐/multi‐shank probe) provided the connections between the probe module and the neural signal amplifier module.

### Optical and Electrical Characterization of the Filter, Probe, and Packaged System

An optimal mixing ratio between the color dye (ABS 473, Exciton, Lockbourne, OH) and the SU‐8 epoxy for sufficient rejection of the excitation light was investigated. 2.8‐µm thick, narrow‐band‐block filter samples with dye concentrations of 2, 4, and 6 wt% were prepared by spin‐coating the filter solution at 3000 rpm for 30 s, pre‐baking the filter layer on a 95 °C hot plate for 90 s, UV exposure on the layer with a 240 mJ cm^−2^ dose, and then post‐baking the exposed filter layer on hot plate at 95 °C for 180 s. The transmittance spectra of the narrow‐band‐block filters in the wavelength range of 400–800 nm were measured using an UV–vis–NIR spectrometer (Cary 5000, Agilent Technologies, Inc., Santa Clara, CA).

An optical power meter (1936‐R, Newport Inc., Irvine, CA) with a photodetector (918D, Newport Inc., Irvine, CA) to measure the light intensity from the tip of the optical fiber for GCaMP excitation and ChrimsonR stimulation was used. The optical fiber was aligned precisely using an XYZ micro‐manipulator, so the emitting tip was placed just above the surface of the photodetector. The measurement of the intensity was conducted in a dark room.

The responsivities of the fabricated photodiode and the filter were measured using 1 µW of monochromatic light at various wavelengths between 400 and 700 nm. For the precise measurement of the responsivity, the characterization was conducted inside a 6‐in. integrating sphere (819C‐SF‐6, Newport Inc., Irvine, CA). A xenon light source (ASB‐XE‐175, Spectral product, Putnam, CT) with a monochromator (CM110, Spectral Products, Putnam, CT) was used to generate light with specific wavelengths. The light generated from the Xenon light source was delivered through an optical fiber into the integrating sphere. The photodiode that was fabricated was mounted on one of the ports of the integrating sphere using a custom‐designed mounting module, and the photocurrent was measured using a source meter (SMU 2450, Tektronix, Inc., Beaverton, OR). At the same time, the intensity of the light delivered to the integrating sphere using the photodetector that was coupled to another port of the integrating sphere was measured.

EIS was conducted to characterize the electrochemical property of the Pt black electrodes using a potentiostat system (PGSTAT101, Metrohm AG, Herisau, Switzerland). A three‐electrode configuration was used for the EIS measurement, and it was comprised of an electrode of the fabricated probe as the working electrode, a platinum wire as the counter electrode, and an Ag/AgCl electrode as the reference electrode. The EIS was conducted in a 1× PBS solution with a frequency range between 1 Hz and 100 kHz.

### Simulation of Fluorescence Detection

Volume of illumination and the “acceptance profile” of the photodiode were calculated using custom ray tracing (Monte Carlo) simulations of photon packets inside a turbid medium^[^ ^49^, ^50^ ^]^ (Figure S7b–d, Supporting Information). Trajectories of randomly generated 108 and 109 photon packets were simulated for the calculation of the volume of illumination and the acceptance cone, respectively, assuming Henyey–Greenstein scattering and absorption taking place inside the brain tissue. For simplicity of simulation, photons were assumed to be monochromatic and reflections and refractions occurring at interfaces between tissue and the other materials were ignored. Parameters used for each simulation are summarized in Table S1 (Supporting Information).

For the calculation of the volume of illumination, trajectories of photon packets generated from the tip of the 50‐µm diameter fiber were tracked and the energy of photons absorbed inside each 10 µm × 10 µm × 10 µm voxel were calculated. Transmitted light intensity (0.3 mW). For the calculation of the acceptance profile of the photodiode, trajectories of photon packets generated from a point source at the center of each voxel were tracked and the energy of photons that pass through the photodiode window (49 × 150 µm) were calculated. The ratio of the sum of the energy of the photons that pass though the photodiode window to the total energy of the photons generated was then calculated, and the ratio was assigned as the detection probability (of a photon generated from a point source located in the specific position).

The volume of detection was then calculated with assumptions that 1) fluorophores uniformly distributed inside the space absorb blue excitation light and generate green fluorescence at a quantum yield of 70%; and 2) fluorophores do not absorb green light. First, the sum of the product of the absorbed energy, the quantum yield, and the detection probability of photon inside each voxel ((Σ Φ(*x*, *y*, *z*)*η*(*x*, *y*, *z*)*p*(*x*, *y*, *z*)) were calculated over the 1 mm × 1 mm × 1 mm volume of simulation to calculate the total energy of green photons that enter the photodiode (Figure S7d, Supporting Information). The detection volume was defined as the volume from which 90% of the photons the photodiode detects are generated. The volume was calculated by counting the number of voxels inside a (sub‐)volume in which sum of the product (Σ Φ(*x*, *y*, *z*)*η*(*x*, *y*, *z*)*p*(*x*, *y*, *z*)) becomes 90% of the total detected energy while adding one voxel with the next largest product (Φ*ηp*) to the sub‐volume at a time.

The red‐light intensity at each point inside the brain tissue was calculated using the same code that was used for the calculation of illumination volume by the blue excitation light (Figure S2, Supporting Information). Trajectories of randomly generated 108 red photon packets were simulated. Parameters used for each simulation are summarized in Table S1 (Supporting Information).

### Animal Preparation

All procedures that involved the use of mice were conducted in compliance with the ethical rules and regulations established by the Animal Care and Use Guidelines of the Korea Institute of Science and Technology (KIST) in Seoul, Korea. Adult male wild mice (C57BL/6; 8–10 weeks old and 25–30 g at AAV virus infection) were used in all of the experiments. The mice were bred in a climate‐controlled animal facility.

A craniotomy was conducted, and the mice were injected to deliver the AAV virus into the target region with 1 µL of AAV1‐Syn‐ or CaMKII*α*‐GCaMP6f‐WPRE‐SV40 (titer: 1.01 × 10^14^ or 1.09 × 10^14^ genome copies mL^−1^ (GC mL^−1^); KIST virus facility, Seoul, Korea) or AAV‐Syn‐ChrimsonR‐tdTomato (titer: 1.53 × 10^13^ GC mL^−1^; KIST virus facility) by a conventional method.^[^ ^51^ ^]^ The GCaMP6f virus was injected into hippocampal CA1 (stereotaxic coordinates from bregma (mm): AP −1.60, ML 1.3, DV −1.35) for recording calcium activity. ChrimsonR was injected into the hippocampal CA3 (stereotaxic coordinates from bregma (mm): AP −1.94, ML 2.10, DV −2.00) region for the circuit study using optical stimulation. For the study of the hippocampal circuit using the multi‐shank probe, CaMKII*α*‐GCaMP6f virus and Syn‐ChrimsonR virus were injected into the CA1 and CA3 regions of the hippocampus, respectively. The mice were subjected to the experiments at least three weeks after the injection of the virus.

### Recording of Ex Vivo Calcium Activity

First, 500‐µm thick coronal brain slices (slide ≈1.7 mm posterior to bregma) were prepared from a mouse that had been anesthetized using 3% isoflurane. The isolated brain was sliced using a vibratome (VT‐1200, Leica, Nusslich, Germany). Before the ex vivo recording, the slices were placed in an interface chamber filled with an artificial cerebrospinal fluid (aCSF) solution and incubated at 36 °C for 1 h. To provide a sufficient penetration depth for the probe, the slice was placed on a 2‐mm thick agarose structure and was held in place using a slice anchor. A fluorescence microscope (Slicescope, Scientifica, East Sussex, UK), in combination with a 10× water immersion objective lens (UMPlanFL N; NA = 0.3, Olympus, Tokyo, Japan), a high‐speed CCD (Neuro CCD, RedShirtImaging, Decatur, GA), a GFP filter (GFP‐ 3035D‐OMF, Semrock, Rochester, NY), and an LED light source (UHP‐Mic‐LED‐460, Prizmatix, Givat‐Shmuel, Israel), was used for the imaging of the calcium signal. The fluorescence probe system was implanted into the brain slice with visual guidance from the fluorescence image using a custom‐built, five‐axis manipulator. To enable the recording of the calcium signals from this region, the photodiode on the probe system was placed directly underneath the sampling region, i.e., the region of interest (ROI), of the microscopic fluorescence image. The GCaMP signal was measured with a CCD camera and the fabricated photodiode at sampling rates of 1 kHz and 60 Hz, respectively, from a hippocampal CA1 region.

To record the change in the calcium signal induced by the electrical stimuli, current pulses (10‐ms long, 50‐Hz two‐paired monophasic pulses with amplitudes ranging from 100 to 200 µA) were applied to the Schaffer Collateral region using a bipolar tungsten electrode (30201, FHC, Bowdoin, ME) in combination with a stimulus isolator (model DS3, Digimeter Ltd., Hertfordshire, UK). To monitor the stimuli applied to the brain slice, an aCSF‐filled glass capillary pipette was placed on CA1, and the stimuli pulse was recorded using a patch‐clamp amplifier (Multiclamp 700B, Molecular Devices, San Jose, CA).

The calcium signals acquired from the CCD were processed with NeuroPlex software (NeuroPlex, RedShirtImaging, Decatur, GA) using a Butterworth low‐pass filter (*f*_co_ = 50 Hz). The signals from the multimodal fluorescence probe were processed using Origin (OriginPro 9.0, OriginLab, Northampton, MA).

### In Vivo Recording

The mice were anesthetized with 0.5% urethane (400 mg kg^−1^, intraperitoneal injection), and then they were placed in the frame of a stereotaxic instrument (Model 940, David Kopf Instruments, Tujunga, CA). The scalp and cranial incisions were made to insert the probe into the target region of the brain. The probe was fixed on a stereotaxic frame with a probe holder to maintain the probe position in the brain during the entire recording session. The probe was implanted into the brain by a robotic stereotaxic manipulator (Neurostar Drill Robot, Neurostar, Tubingen, Germany) at an insertion rate of 1 mm min^−1^. The probe could be inserted into the brain without buckling of the shank (rate of successful implantation = 100%). At the target region, spontaneous activities were recorded for 30 min, and the calcium signal was measured using 150 µW of 475‐nm excitation light. For the measurement of the changes in the calcium signal and the electrical signals, the probe was implanted into CA1 (recording coordinates from bregma (mm): AP −1.7, ML 1.5, DV −1.7) and CA3 (recording coordinates from bregma (mm): AP −1.8, ML 2, DV −2.3). Electrical signals were acquired using an Intan system (RHD3216 in combination with RHD2000 USB interface board, Intan Technology, Los Angeles, CA) at a sampling rate of 30 k s^–1^ per channel. A 0.3–6 kHz bandpass filter was applied to the recorded signal. Optical signals were recorded with a sampling frequency of 60 Hz using a source meter and/or a multimeter (2450 and DMM6500, Tektronix, Inc., Beaverton OR). The multi‐shank probe had two types of LEDs to study the neural circuit, i.e., one for the excitation light and the other for the optical stimulation. A DC power analyzer (N6705C, Keysight Technologies, Santa Rosa, CA) precisely controlled the light pulses to the LED for optical stimulation. The stimulation light at 625 nm of wavelength was illuminated with 100 µW, and the frequency of the square wave was 0.5 Hz with a 25% duty cycle.

### Signal Analysis

For the first step of the analysis, the fluorescence signal using Origin software was filtered and normalized. A Butterworth high‐pass filter (*f*_cutoff_ = 0.05–0.1 Hz, second‐order) was used to calculate the baseline from the entire fluorescence trace and to attenuate the fluctuation of the baseline. An additional low‐pass Butterworth filter (*f*_cutoff_ = 5 Hz, second‐order) was used to reduce the noisy fluctuations in the calcium signal further. After the baseline was corrected and the noise was attenuated, the normalized fluorescence signal (Δ*F*/*F*_0_) was calculated using the following equation: Δ*F*/*F*_0_ = (*F*(*t*) − *F*(0))/*F*(0). After normalization of the signal, the MLspike algorithm^[^ ^52^ ^]^ was used to perform a maximum likelihood estimation of the spike trains underlying each fluorescence trace in the in vivo datasets.

The measured electrical signals were analyzed using Origin, Spike2 (Version 7.09, Cambridge Electronic Design, Limited, Cambridge, England) and MATLAB (Version 2019a, MathWorks, Natick, MA). First, spikes in the electrical signal recorded from each channel were sorted and analyzed using Spike2. The waveform and the timestamp information of each sorted unit were extracted from Spike2. Using MATLAB, the locations of the neurons were estimated via trilateration,^[^ ^34^ ^]^ using the coordinates of the electrode as the known centers and the relative amplitude of the recorded spikes as the ranges. Autocorrelograms were calculated in Origin for the quantitative analysis of the correlation between the fluorescence signal and the electrical signal. Then, the correlation between the signals estimated from the fluorescence and the spikes obtained from the sorting of the electrical data was calculated. The SNR of the fluorescence signals and the electrical signals were calculated using the following equation: SNR = max(peak)/3·SD_baseline or background noise_. Statistical analyses of the data were performed in Excel and Origin software using a two‐tailed, unpaired *t*‐test method to compare the two groups.

### Histology

After the in vivo recording experiments, the mouse was perfused transcardially with physiological saline and then infused with fixative (4% paraformaldehyde in 0.1 m PBS). Subsequently, it was fixed in the same fixative overnight. Then, the brain of the mouse was immersed in 30% sucrose in 0.1 m PBS for 24 h. Afterward, the brain was cut into 50‐µm coronal sections using a cryostat microtome. The brain sections were washed for 5 min in 0.1 m PBS, after which the brain sections were incubated for 10 min at room temperature with 4′, 6‐diamidino‐2‐phenylindole (DAPI; 1:1000, D1306, Invitrogen) in 0.1 m PBS. After washing the sections three times for 5 min in 0.1 m PBS, the sections were mounted on a coverslide with a mounting medium. To confirm the probe track, sections were imaged through 5× and 10× objectives on a confocal microscope (LSM 700, Carl Zeiss, Oberkochen, Germany) with the identical scope setup.

## Conflict of Interest

The authors declare no conflict of interest.

## Supporting information

Supporting Information

## Data Availability

Research data are not shared.
