# Supplementary material for: A Multimodal Multi‐Shank Fluorescence Neural Probe for Cell‐Type‐Specific Electrophysiology in Multiple Regions across a Neural Circuit
Source: Adv Sci (Weinh). 2021 Nov 19;9(2):2103564. doi: 10.1002/advs.202103564 (PMC8805556; doi:10.1002/advs.202103564)
Supplement: Supplementary file 1 — Supporting Information [file ADVS-9-2103564-s001.pdf]

WILEY-VCH

ADVANCED SCIENCE
Open Access

Supporting Information

for *Adv. Sci.*, DOI: 10.1002/advs.202103564

A multimodal multi-shank fluorescence neural probe for cell-type-specific electrophysiology in multiple regions across a neural circuit

*Namsun Chou, Hyogeun Shin, Kanghwan Kim, Uikyu Chae, Minsu Jang, Ui-Jin Jeong, Kyeong-Seob Hwang, Bumjun Yi, Seung Eun Lee, Jiwan Woo, Yakdol Cho, Changhyuk Lee, Bradley J Baker, Soo-Jin Oh, Min-Ho Nam, Nakwon Choi, and Il-Joo Cho***

* Corresponding author.

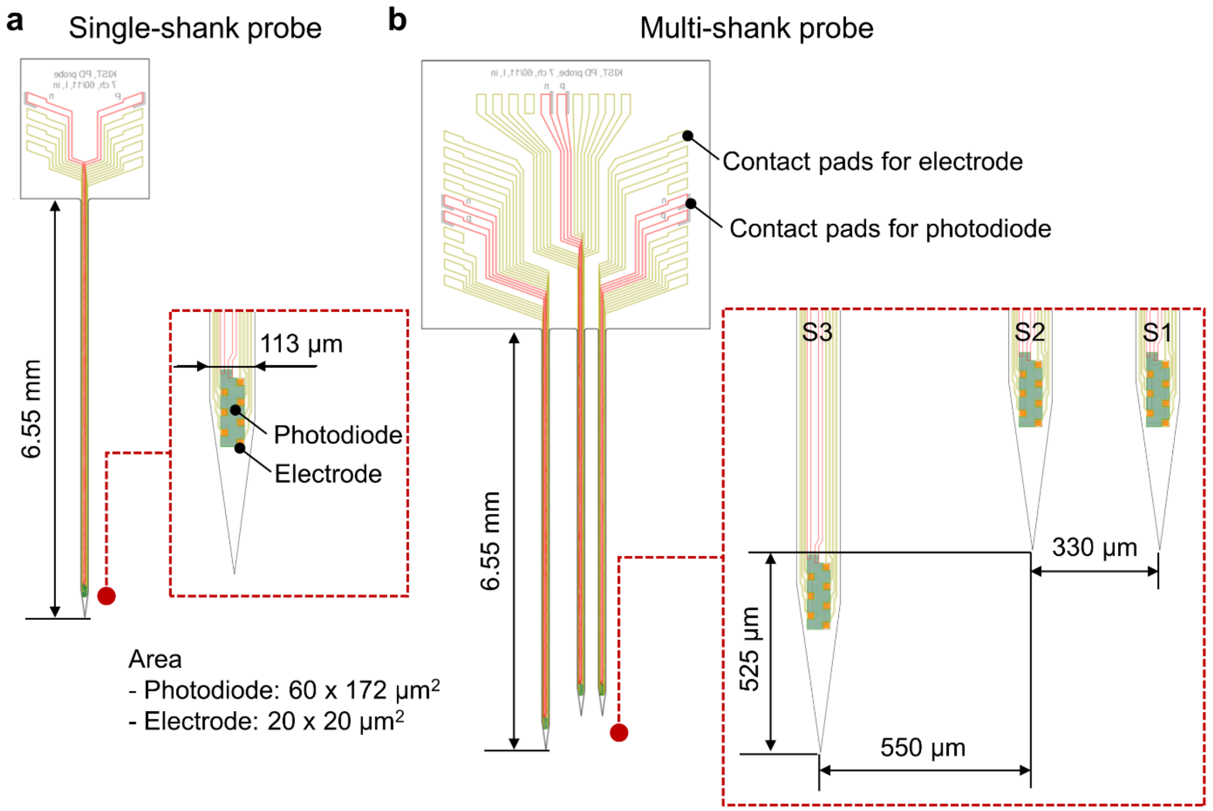

Figure S1. Dimensions of a) the single-shank and b) the multi-shank probe

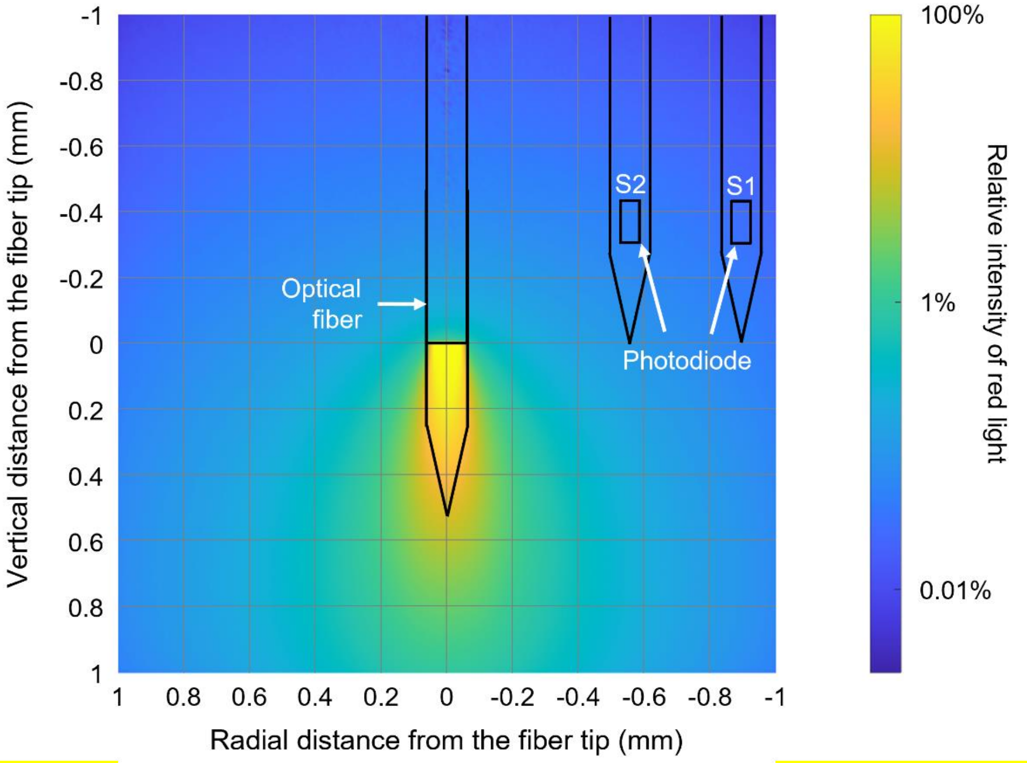

Figure S2. Simulation result of the relative intensity of scattered red light from the distance of optical fiber tip. The intensity of red light at the fiber tip is 3 mW/mm2. The position of optical fiber, shank and photodiode are indicated with black outlines.

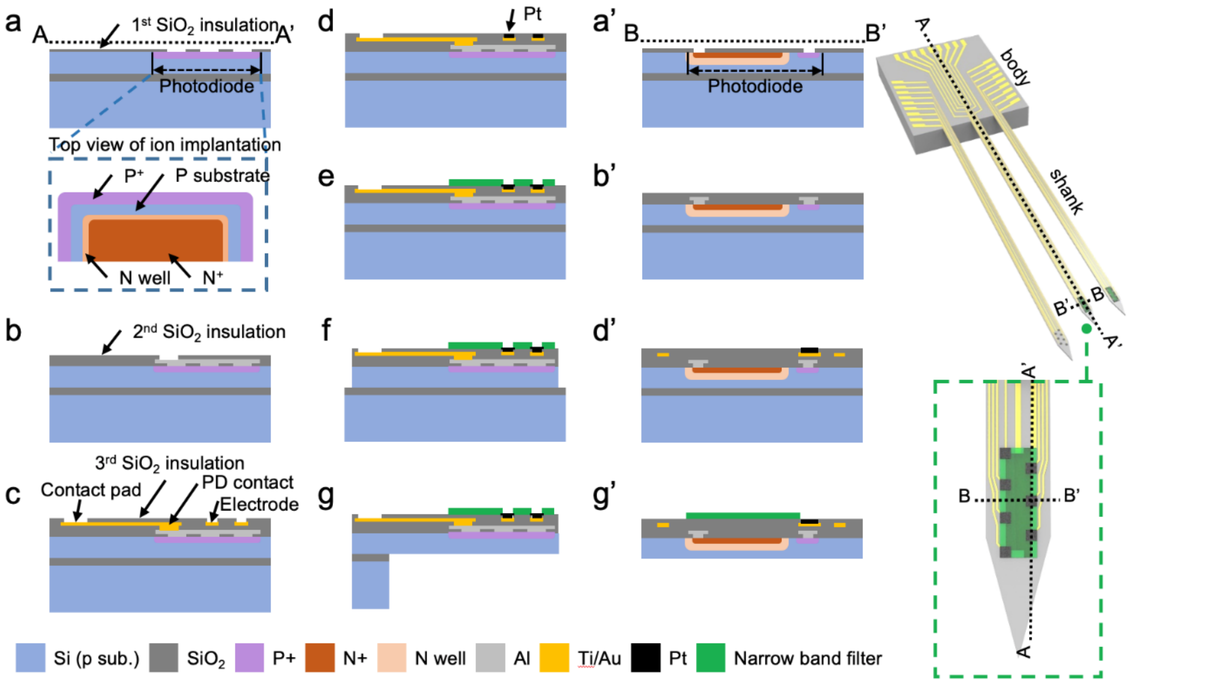

Figure S3. Fabrication procedure of the multimodal fluorescence neural probe system. A cross-sectional view of A-A a to g) used to present the detailed fabrication steps at the point of view for electrode-photodiode location. In addition, A cross-sectional view of B-B a', b', d' and g') shows for the additional information at the viewpoint of p-n junction and electrode. a and a') n well, n^+ and p^+ ion implantation on the surface of the top Si substrate for a PN junction photodiode. The inset image of a top view of ion implantation presents the patterns of n well, n^+ and p^+. Next, a 1st SiO_2 layer was deposited and patterned for the insulation and contact opening of the photodiode. b and b') Sputtering and etching of the Al for an electrical connection of the photodiode. Then, a 2nd SiO_2 layer deposition and patterning for the insulation and the via connection between Al and Ti/Au signal lines. c) Sputtering and patterning of the Ti/Au layer for signal lines and electrodes. Deposition and patterning of a 3rd SiO_2 layer for the insulation of signal lines and the opening of electrodes and pads. d and d') Sputtering and lift-off of the Pt layer to achieve the adhesion between Au and Pt black. e) Lithographically patterning of the narrow-band-block filter using a photo-definable filter based on SU8. f, g and g) Isolation of a shank and a body by dry etching of top and bottom Si substrate until exposing the buried SiO_2 layer using a deep reactive ion etching (DRIE).

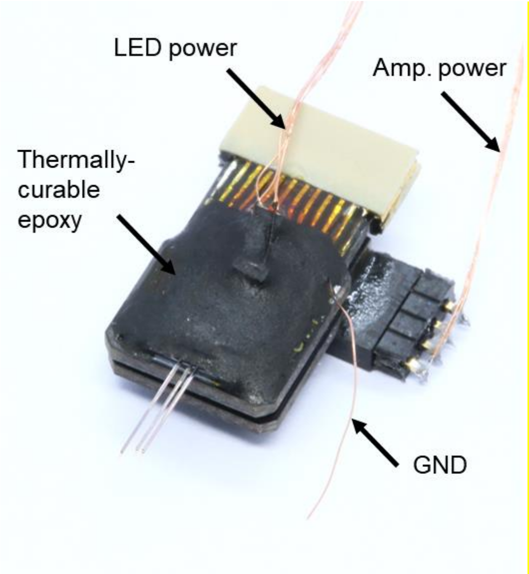

Figure S4. Image of the packaged fluorescence neural probe.

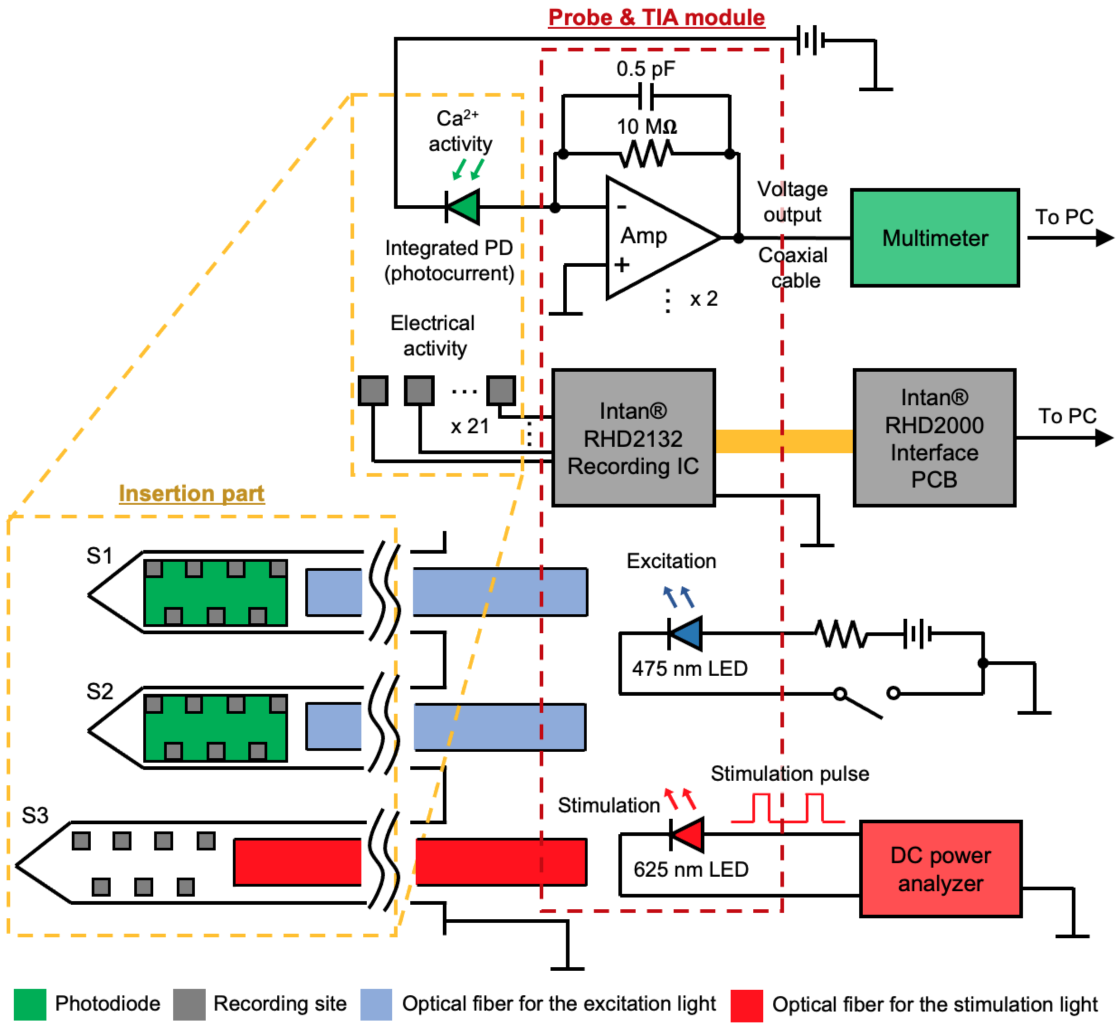

Figure S5. Schematic illustration of the circuit diagram. A box with yellow-dashed line presents the insertion part of multi-shank probe, consisting of three shanks with photodiodes, electrodes and optical fibers. S1 and S2 shank provide optical and electrical recording and S3 provides optical stimulation and electrical recording. A box with red-dashed line indicates the probe & TIA module, integrating with two TIAs, an Intan recording IC and two LEDs. The transimpedance amplifier converts the photocurrent to voltage output with 10^7 gain by, and then the multi-output voltage reads and transfers the optical output signals. The Intan interface records the electrical signals and then transfers to the PC. A battery with a manual switch provides the power of LEDs for the excitation light. The DC power analyzer supplies and controls the red-light pulses of an optical stimulation for neural circuit study.

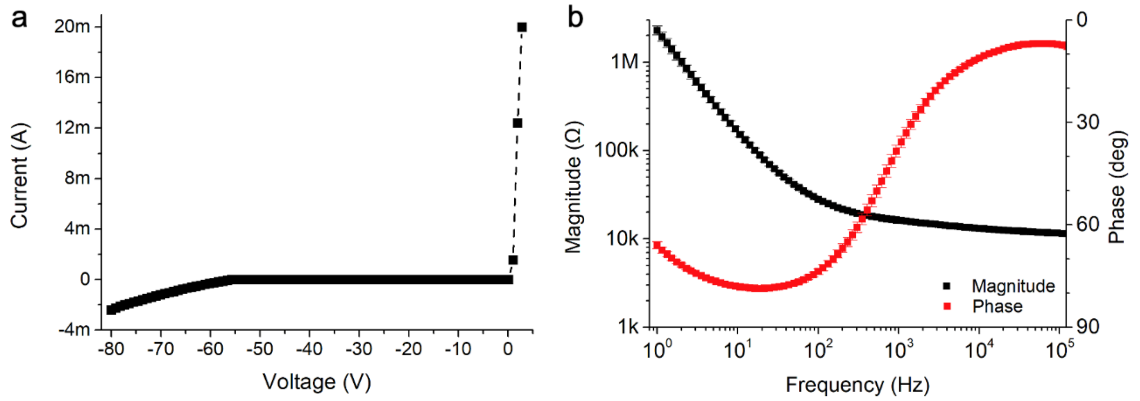

Figure S6. Characterization of the photodiode and the Pt black coated electrodes integrated on the fluorescence probe. a) A I-V curve of the photodiode in a range of bias voltage from 5 to -80 V in dark cage. b) The electrochemical impedance spectroscopy (EIS) in a range from 0 to 100 kHz at four electrodes from two electrode arrays.

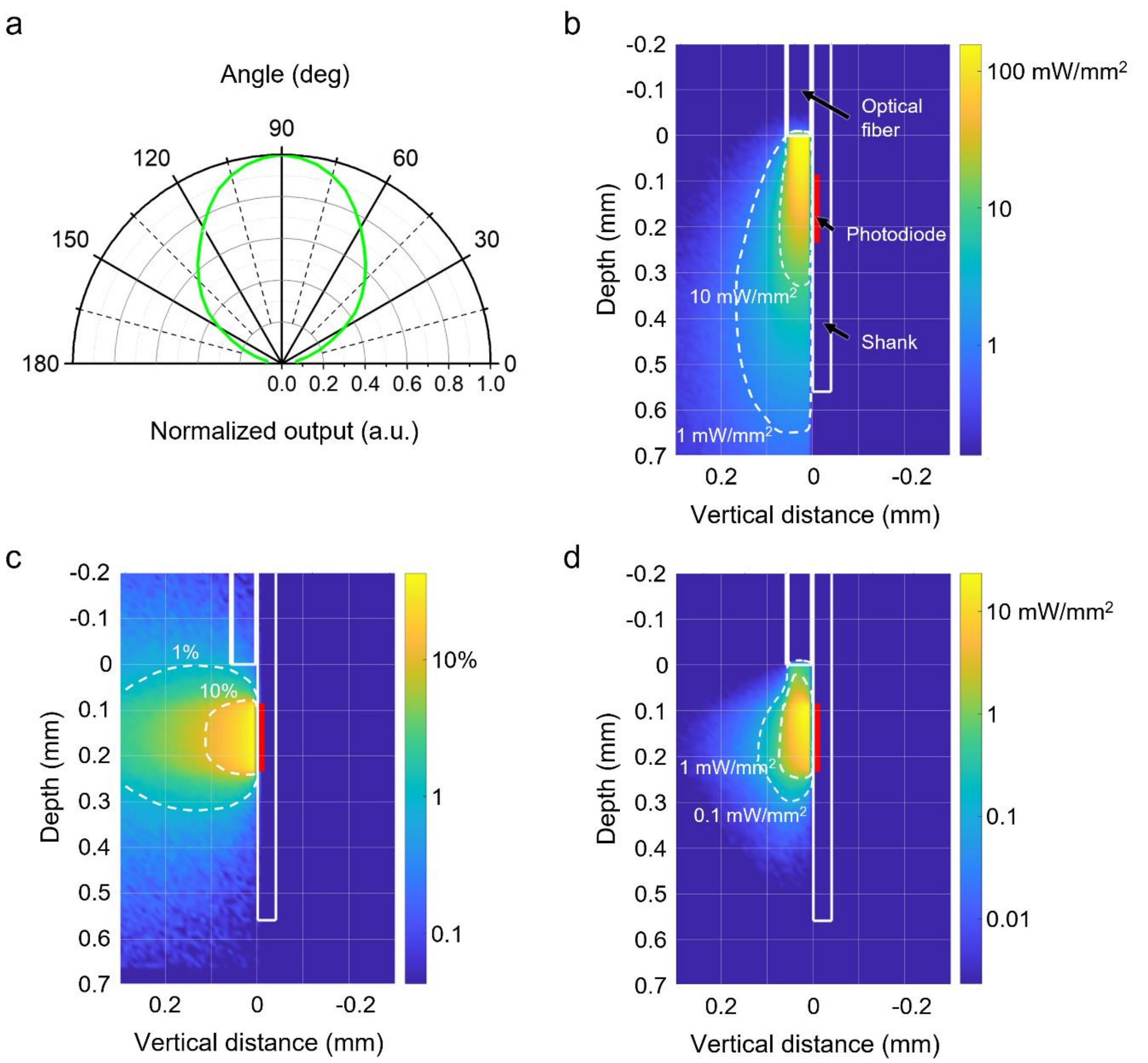

Figure S7. a) Angular acceptance profile of the photodiode integrated on the neural probe, measuring using a 520-nm wavelength light source (Light intensity: 400 nW/mm^2). b) Simulated intensity profile of the 0.3 mW blue excitation light from the integrated fiber tip. c) Simulated acceptance profile of photodiode in the brain tissue. d) Simulated profile of the captured fluorescence by the integrated photodiode. The density profile was calculated using the simulation results presented (b) and (c).

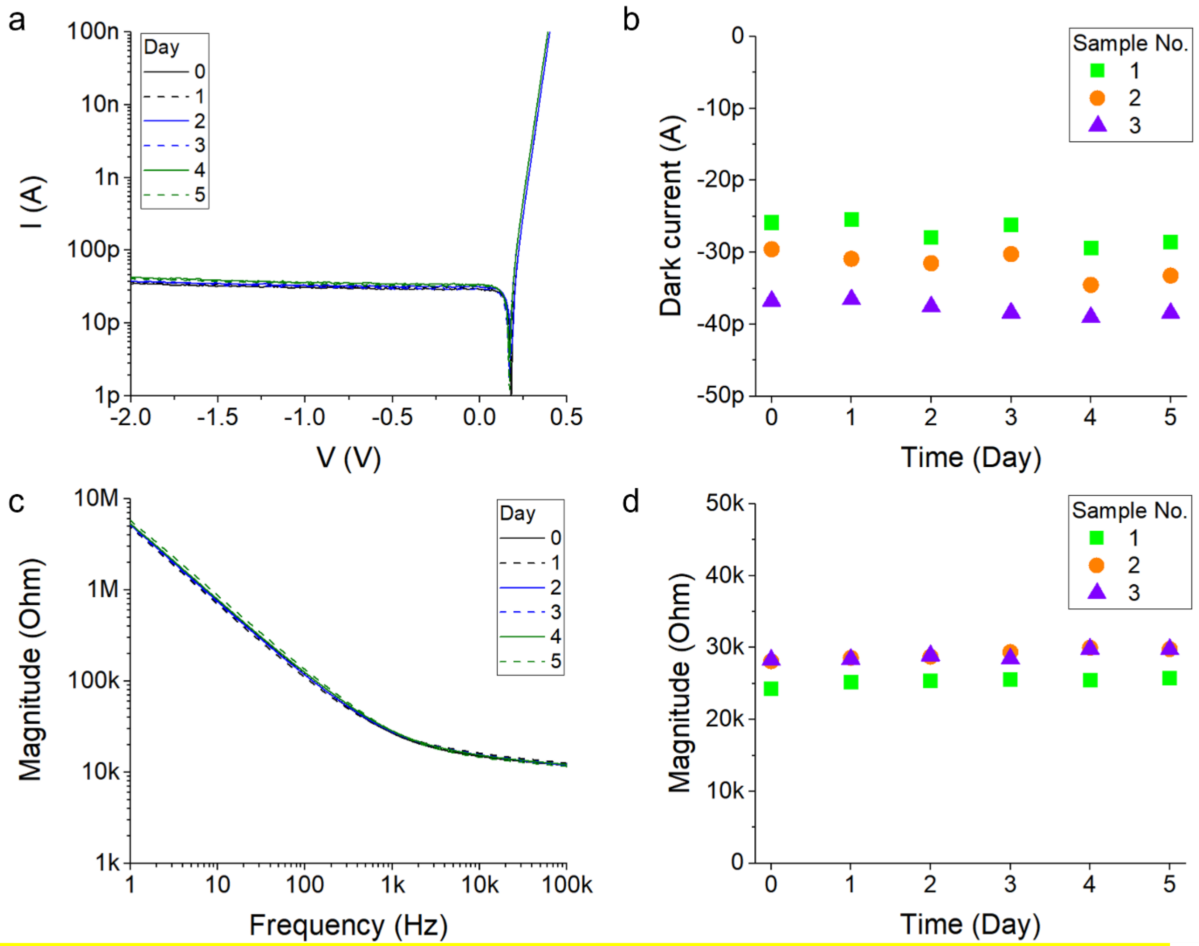

Figure S8. Results of accelerated in vitro aging test of the photodiode and electrodes for the characterization of long-term stability. The electrochemical impedance of the electrode and the I-V characteristics of the photodiode inside a 67 °C 1x PBS solution were measured once a day for five days. (a) I-V curves of the photodiodes in a bias voltage range from 0.5 to -2 V. (b) Dark current of photodiodes at -0.5 V of reverse bias voltage (n=3). (c) Electrochemical impedance spectroscopy (EIS) of electrodes from 1 to 100 kHz. (d) Impedance magnitude of electrodes at 1 kHz (n=3).

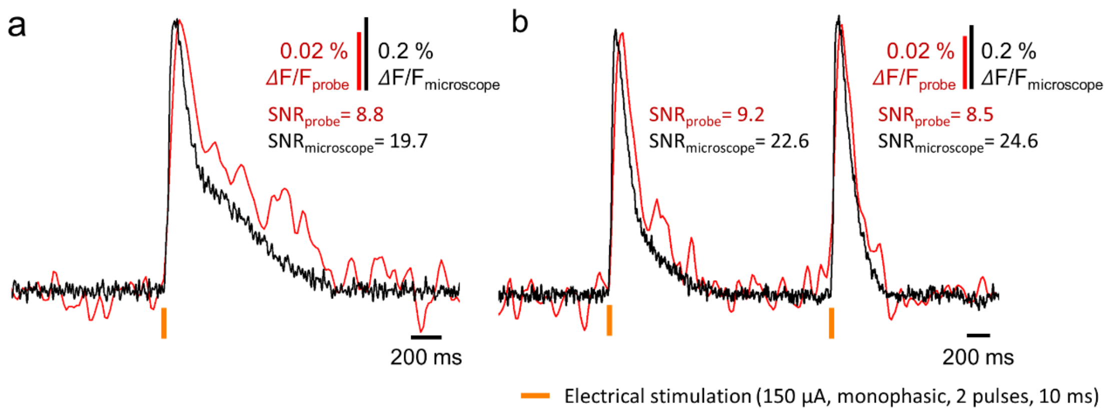

Figure S9. An overlaid transient plot of the electrical stimuli induced calcium activity from the fluorescence neural probe (red) and the fluorescence microscopy (black). Recording results of a single a) and double b) of electrical stimuli in a single recording trial.

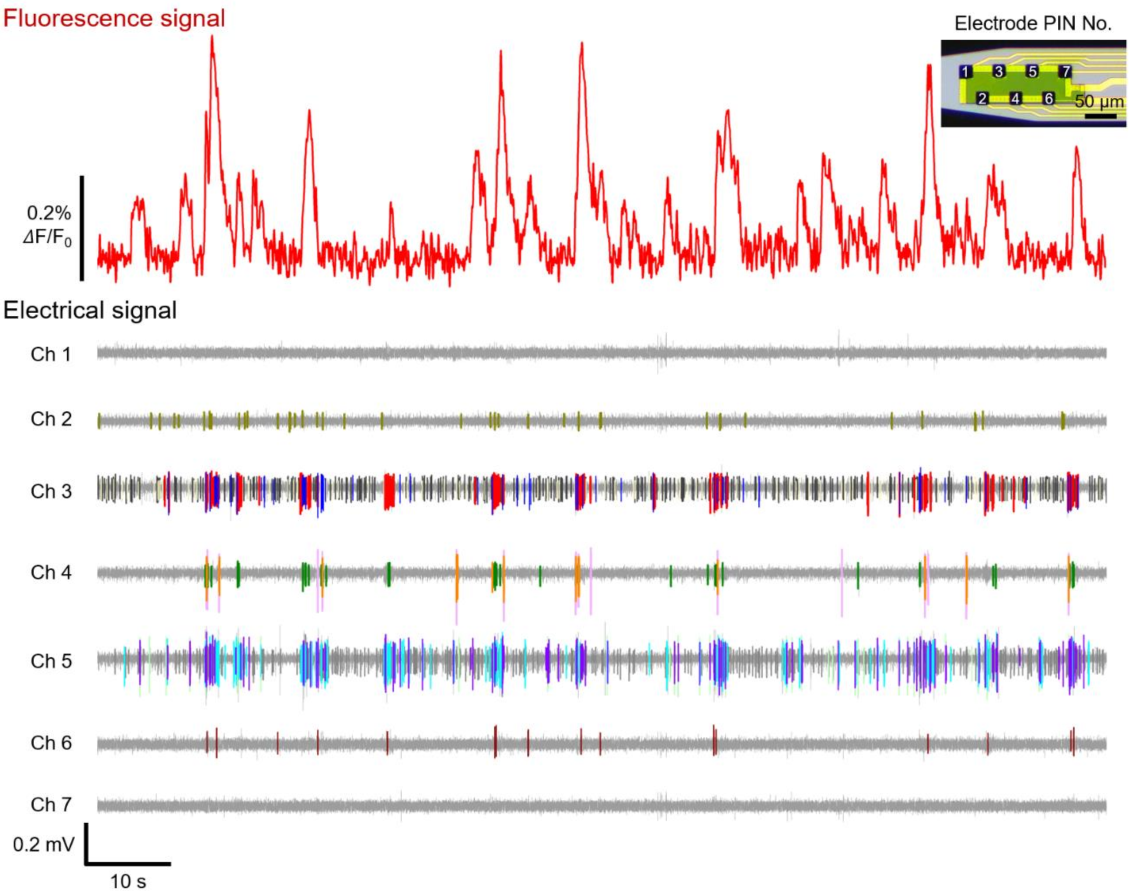

Figure S10. Recording result of fluorescence and electrical signals in all channels of the single-shank probe. After signal sorting of the electrical signals measured in all channels, the electrical spikes contributing to the calcium change were analyzed and identified.

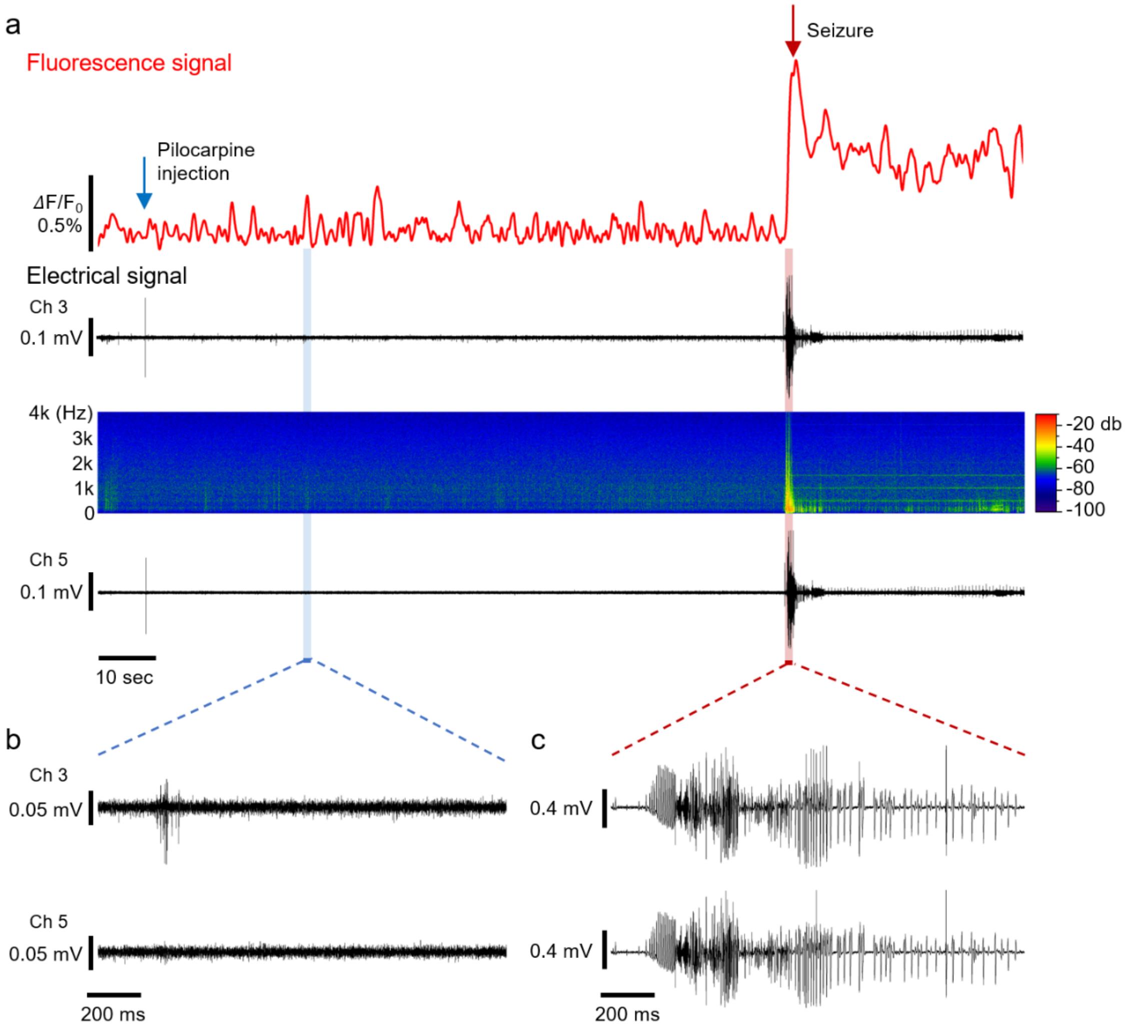

Figure S11. In vivo recording results of a pharmacological stimuli-induced seizure. To develop seizure, mice with AAV-Syn-GCaMP6f expression in CA1 administered an intraperitoneal (i.p.) injection of pilocarpine at a dose of 300 mg/kg body weight. The optical and electrical signals were monitored at the CA1 hippocampus from before i.p. Injection to after seizure. a) Transient plot and heat map of the recorded fluorescence and electrical activity in the CA1 region. The amplitude of fluorescence and electrical signals were significantly increased at an evoked time of pilocarpine-induced seizure activity (red arrow). The zoom-in view of the electrical signals reveals the series of changes observed b) before and c) after the seizure.

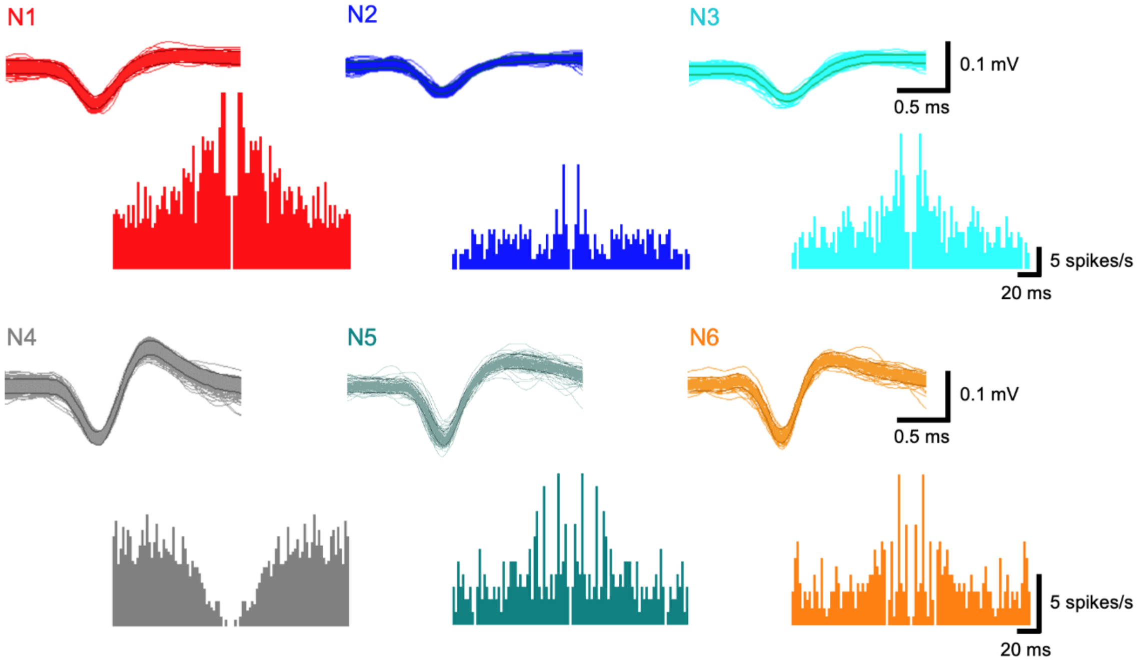

Figure S12. The waveform and autocorrelogram of the sorted spikes at the recorded signal form CamKIIα-GCaMP6f expressing mouse. Spikes were plotted and analyzed using the result from the sorted N1 to N6 neurons shown in Figure 5.

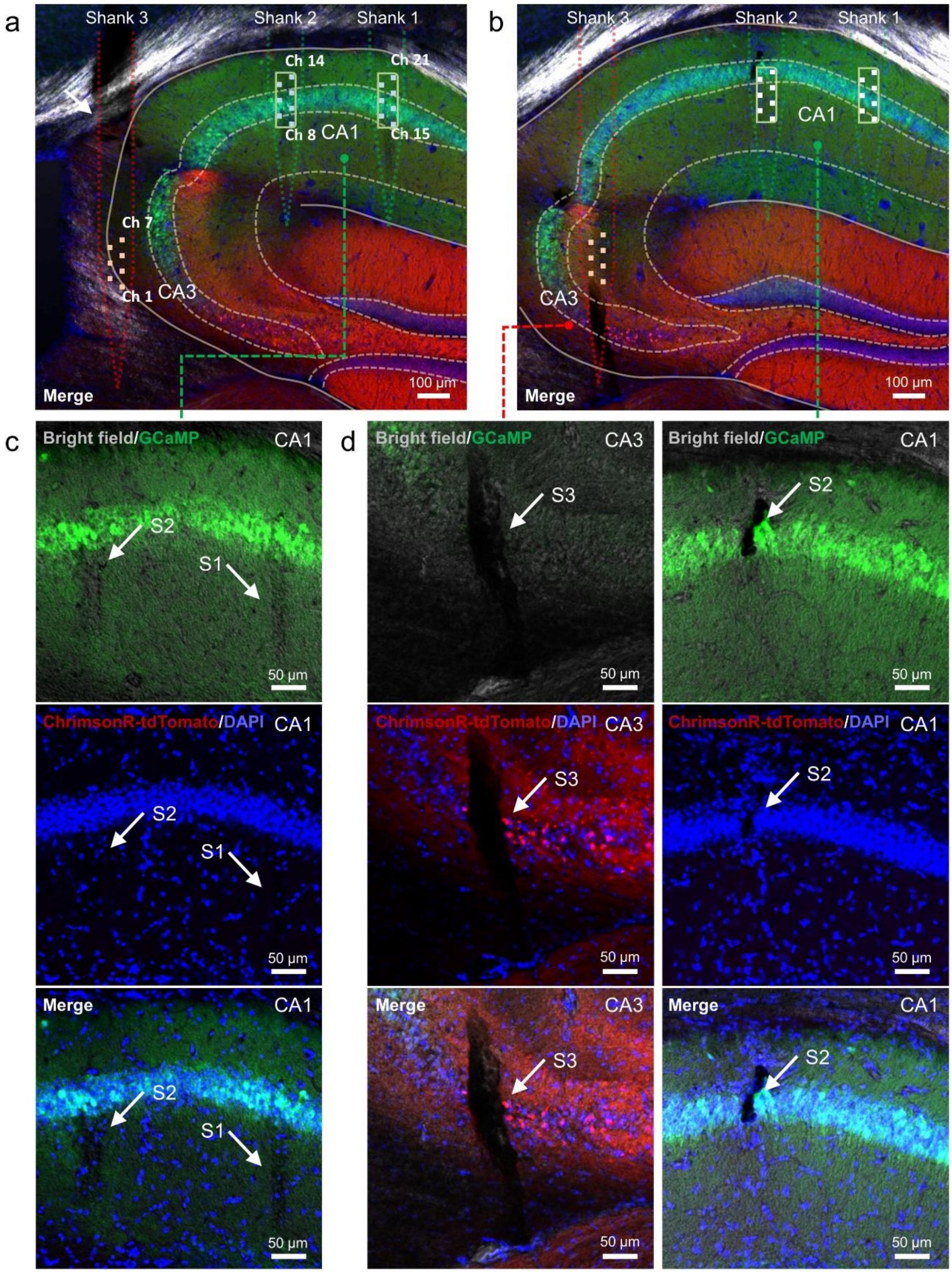

Figure S13. Confocal images of the brain slice after probe insertion at the hippocampal circuit from an AAV-CamKIIa-GCaMP6f (CA1) and Syn-ChrimsonR-tdTomato (CA3) expressed mouse. DAPI immunostaining of nucleus (blue) on same brain slice. Fluorescent images successfully show insertion region of multi-shank fluorescence neural probe using the tissue scar at brain slice. The coronal-sectioned brain slices were prepared with a thickness of 50 um. The brain slice of image (b) is 150 µm posterior from slice of (a) The GCaMP and ChrimsonR was strongly expressed at CA1 and CA3 region without significant overlap (a and b). The recording shank (Shank 1 and 2) and stimulation shank (Shank 3) was successfully targeted at CA1 and CA3 region (c and d).

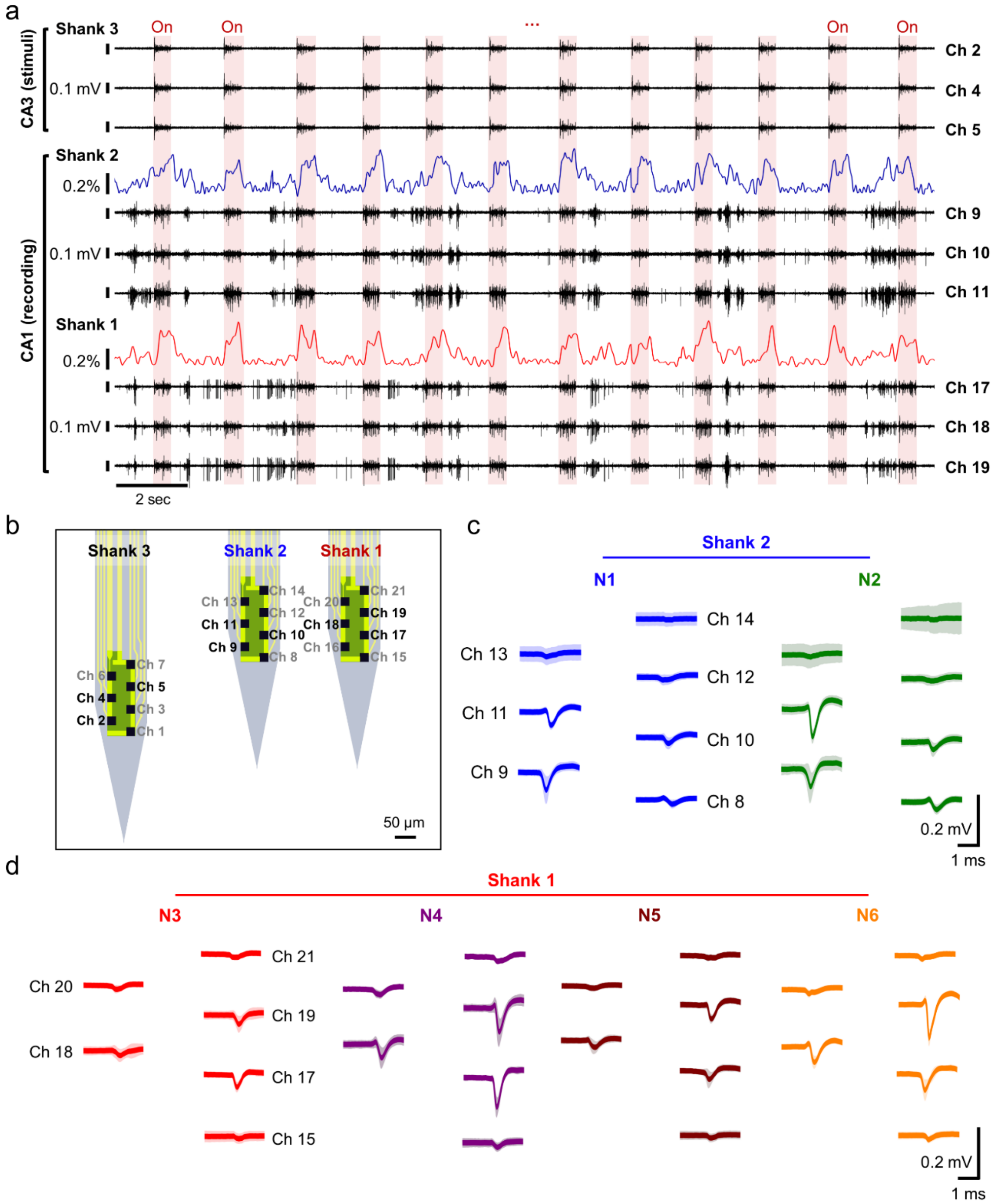

Figure S14. The recorded fluorescence and electrical signals from the hippocampal CA3-CA1 circuit using the multi-shank probe. a) Transient plot of electrical and calcium signals over fluorescence stimulation (0.5 Hz, 25% duty cycle; red background indicates the onset of light). b) The schematic illustration of the tip of the fluorescence neural probe. (c and d) The short trace of each neuron from Shank 2 (N1 and N2; c) and Shank 3 (N3 to N6; d) was overlaid at the timing of sorted spikes.

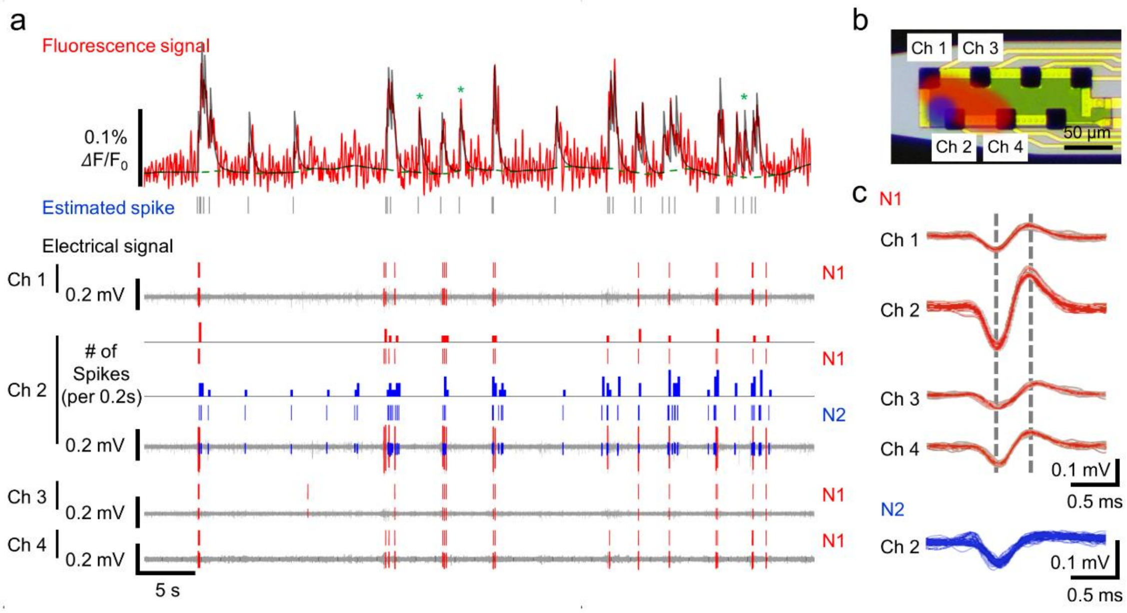

Figure S15. Matching and analysis between the in-vivo fluorescence and electrical signals using the MLspike algorithm. a) The transient plot of fluorescence and electrical signals from the excitatory neuronal activities in the CA1 region of AAV1-CaMKII**α**-GCaMP6f expressing mouse. Electrical spikes were sorted and were classified for the identification of calcium contributed spikes. In addition, the calcium signal and estimated electrical spikes from the measured calcium activities were evaluated by the MLspike algorithm. Independent changes in calcium without a change in electrical neural signals were labeled as a green star. b) The illustrated plot of the estimated location of two neurons using the identified neuron activity contributing to the calcium change. c) Waveform from two identified neurons. Spikes of the N1 neuron were recorded from channel 1 to 4.

Table S1. Scattering / absorption parameters utilized for Monte Carlo simulation of light propagation inside brain tissue

	**Excitation light (Illuminated volume calculation)**[1]	**Emission light (Acceptance cone calculation)**[2]	**Stimulation light (Red light intensity calculation)**[1]
**Wavelength (λ, nm)**	475	510	633
**Absorption coefficient (μ_a, mm^{-1})**	0.38	0.04	0.07
**Scattering coefficient (μ_s, mm^{-1})**	11.1	10.6	9.1
**Asymmetric parameter (g)**	0.89	0.88	0.89

References

[1] J. D. Johansson, *J. Biomed. Optics*, **2010**, 15, 5.

[2] A. N. Yaroslavsky, P. C. Schulze, I. V. Yaroslavsky, R. Schober, F. Ulrich and H-.J. Schwarzmaier, *Phys. Med. Biol.*, **2002**, 47, 12.
